# miR-147a targets ZEB2 to regulate ox-LDL-induced monocyte adherence to HUVECs, atherosclerotic plaque formation, and stability in atherosclerosis

**DOI:** 10.1016/j.jbc.2023.104657

**Published:** 2023-03-29

**Authors:** Fengying Chen, Yuzhen Ning, Jingying Liu, Ming Lian, Juanjuan Wang, Hongwei Dan

**Affiliations:** Emergency Department, The Affiliated Hospital of Inner Mongolia Medical University, Hohhot, Inner Mongolia, China

**Keywords:** miR-147a, ZEB2, monocyte adherence, M1/M2 macrophage polarization, atherosclerotic plaques

## Abstract

The mechanisms underlying atherosclerosis (AS) that seriously affect human health, such as those involved in endothelial cell injury and monocyte/macrophage aggregation and infiltration, have not been fully elucidated. To investigate these processes, we established human umbilical vein endothelial cells (HUVECs) injured by oxidized low-density lipoprotein (ox-LDL) to mimic AS *in vitro*. Apolipoprotein E knockout (ApoE^−/−^) C57BL/6 mice were fed with a high-cholesterol diet to establish an AS model *in vivo*. We detected HUVEC apoptosis, and apoptosis-related proteins by 3-[4,5-dimethylthiazol-2-yl]-2,5 diphenyl tetrazolium bromide and lactate dehydrogenase, flow cytometry, and Western blot assays, respectively, and we observed monocytes (THP-1 cells) adhering to HUVECs. Furthermore, miR-147a and its downstream target gene ZEB2 (zinc finger E-box binding homeobox 2) were predicted by bioinformatics analysis to be involved in AS, and their correlation was confirmed by several experiments. We determined the localization of miR-147a and ZEB2 within macrophages of AS mice by *in situ* hybridization and immunofluorescence. Atherosclerotic plaques in whole aortas were detected by histology observation. miR-147a attenuated adherence of monocytes to HUVECs and the upregulation of mononuclear chemotactic adhesion receptors in THP-1 cells induced by ox-LDL-injured HUVEC supernatants through directly downregulating ZEB2 levels. Moreover, miR-147a influenced M1/M2 macrophage polarization from THP-1 cells and the roles of their supernatants (THP-1 cells) in HUVEC apoptosis. miR-147a targeted ZEB2 to impact lipid accumulation and atherosclerotic plaque formation through regulating M1/M2 polarization and macrophage adhesion in AS mice. In summary, miR-147a attenuates ox-LDL-induced adherence of monocytes to HUVECs and modulates atherosclerotic plaque formation and stability through targeting ZEB2 during AS.

Atherosclerosis (AS) is a chronic disease, mainly attributed to hypertension, hypercholesterolemia, and so on, which is characterized by progressive lipid deposition in the arterial wall, plaque induction, and possible further progression to the state of so-called unstable plaque (1). Destabilization of plaques causes their ruptures, followed by thrombosis that can result in dangerous outcomes, covering acute coronary syndrome and stroke (2). Therefore, resisting lipid deposition and plaque induction and stabilizing AS plaque have been prevailing issues to be addressed in the field of cardiovascular and cerebrovascular diseases.

Among various pathological factors, the role of endothelial cell injury and monocyte/macrophage aggregation and infiltration in AS development should not be underestimated. The two factors usually interact with each other rather than changing independently in pathology. Endothelial cell injury can cause monocytes in blood to aggregate in vascular endothelium and migrate to the subendometrium to form macrophages. Macrophage infiltration can also trigger vascular endothelial cell damage, and endothelial dysfunction is a precursor factor for AS, during which the oxidized low-density lipoprotein (ox-LDL) is presently considered to exert a critical effect (3). Ox-LDL engenders endothelial dysfunction by upregulating adhesion molecules and recruiting the monocytes into the subendothelial space to form macrophages that further engulf lipid to form lipid stripes and atherosclerotic plaques–AS characteristic lesions (3, 4). Consequently, it is important to block macrophage infiltration in arterial wall and the formation of foam cells, lipid stripes, and atherosclerotic plaques, thereby preventing ox-LDL-induced vascular endothelial cell injury and the release of chemotactic adhesion molecules after injury to reduce the resulting monocyte adhesion to vascular endothelium.

miRNAs are a type of small noncoding RNAs with fewer than 22 nucleotides that inversely modulate transcription by repressing protein translation or degradation. Accumulating evidence has indicated that abnormal miRNA expression can cause different cardiovascular disorders, such as AS (5, 6). miRNAs are involved in the formation and regression of AS lesion, reflecting the potential diagnostic, prognostic, and therapeutic roles of miRNAs in AS (7). The atherogenic stimulation of monocytes and macrophages induced by ox-LDL also alters the miRNA expression profile, including the expressions of miR-155 and miR-146a, which, in turn, affects lipid uptake and inflammatory cytokine secretion (8). However, it is still unclear whether and which miRNA could impact ox-LDL-induced vascular endothelial cell injury and the monocyte adhesion to vascular endothelium during AS.

Zinc finger E-box binding homeobox (ZEB) 2 makes profound impacts on cancer cells, cancer stem cells, and nervous system development, which is a risk factor for vascular diseases such as AS (9, 10, 11). Ma *et al.* (12) disclosed the multifaceted action mechanisms of the ZEB2-associated coronary artery disease (CAD) risk SNPs. Cheng *et al.* (13) reported that ZEB2 is a new CAD genome-wide association study gene that affects plaque vulnerability *via* direct effects on the epigenome. The findings reported by Gladka *et al.* (14) also revealed that ZEB2 is expressed in injured cardiomyocytes and is a beneficial factor during ischemic injury. Still, the exact mechanisms of ZEB2 in AS await to be expounded.

In this study, we capitalized on human umbilical vein endothelial cells (HUVECs) and a human monocyte cell line, THP-1, to explore the role of ox-LDL in vascular endothelial cell injury and monocyte adhesion to vascular endothelium, as well as inhibition of plaque formation and enhancement of plaque stability. In addition, further investigation on the underlying mechanism involving miRNA may be conducive to anti-AS.

## Results

### Ox-LDL injured HUVECs and promoted its adhesion to THP-1 cells and expressions of adhesion molecules

**Figure 1:**
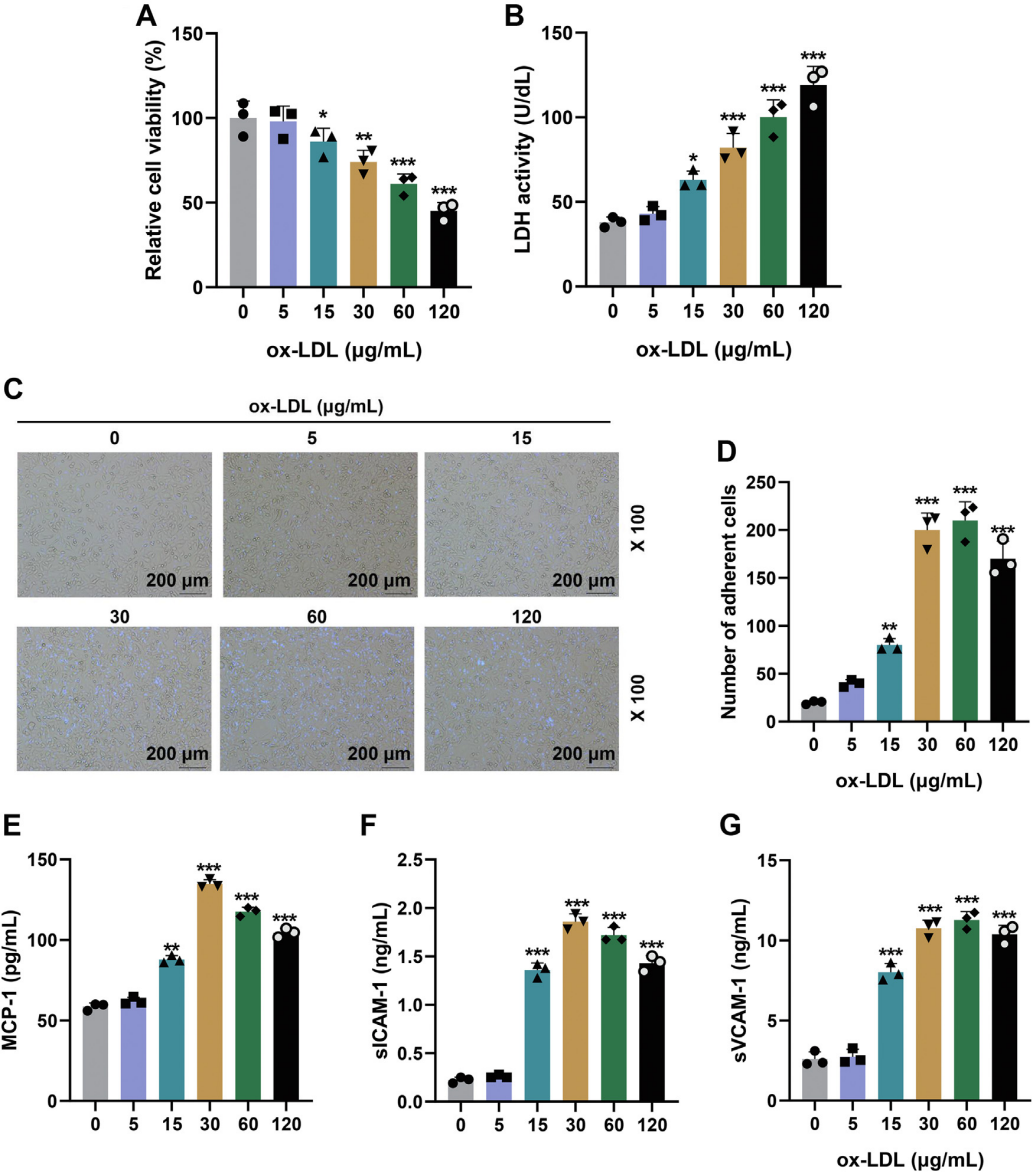
**Ox-LDL injured HUVECs and promoted its adhesion to THP-1 cells and expressions of adhesion molecules.***A*, cell viability was detected by MTT assay in HUVECs treated with ox-LDL at different concentrations. *B*, LDH activity in HUVECs among groups. *C*, Ox-LDL promoted adhesion between HUVECs and THP-1 cells. *D*, quantification of adhesion conducted by counting the number of adherent THP-1 cells (*violet*) per field. *E*–*G*, the expressions of adhesion molecules (MCP-1, sICAM-1, and sVCAM-1) in supernatants of HUVECs after culture with ox-LDL were detected by ELISA. ∗*p* < 0.05, ∗∗*p* < 0.01, ∗∗∗*p* < 0.001 *versus* 0. The data were expressed as mean ± SD. The one-way ANOVA was used to compare multiple groups, followed by post hoc Dunnett’s test. Experiments in this figure were repeated three times, and similar results were obtained. HUVEC, human umbilical vein endothelial cell; LDH, lactate dehydrogenase; MCP-1, monocyte chemoattractant protein 1; MTT, 3-[4,5-dimethylthiazol-2-yl]-2,5 diphenyl tetrazolium bromide; Ox-LDL, oxidized low-density lipoprotein; sICAM-1, soluble intercellular cell adhesion molecule-1; sVCAM-1, soluble vascular cellular adhesion molecule-1.

In addition to 5 μg/ml ox-LDL, ox-LDL at other concentrations dose-dependently suppressed HUVEC viability and enhanced lactate dehydrogenase (LDH) release compared with 0 μg/ml ox-LDL (Fig. 1, *A* and *B*, *p* < 0.05). From Figure 1, *C* and *D*, unstained cells in low layers were HUVECs, whereas the cells stained with the Hoechst dye were THP-1 cells. HUVECs showed monocyte adherence after ox-LDL treatment (Fig. 1, *C* and *D*, *p* < 0.01). Moreover, except for 5 μg/ml ox-LDL, other concentrations of ox-LDL increased the levels of monocyte chemoattractant protein 1 (MCP-1), soluble intercellular cell adhesion molecule-1 (sICAM-1), and soluble vascular cellular adhesion molecule-1 (sVCAM-1) in HUVEC cultural supernatants (Fig. 1, *E*–*G*, *p* < 0.01).

### The supernatants of ox-LDL-treated HUVECs increased mononuclear chemotactic adhesion receptor levels and decreased miR-147a level in THP-1 cells

**Figure 2:**
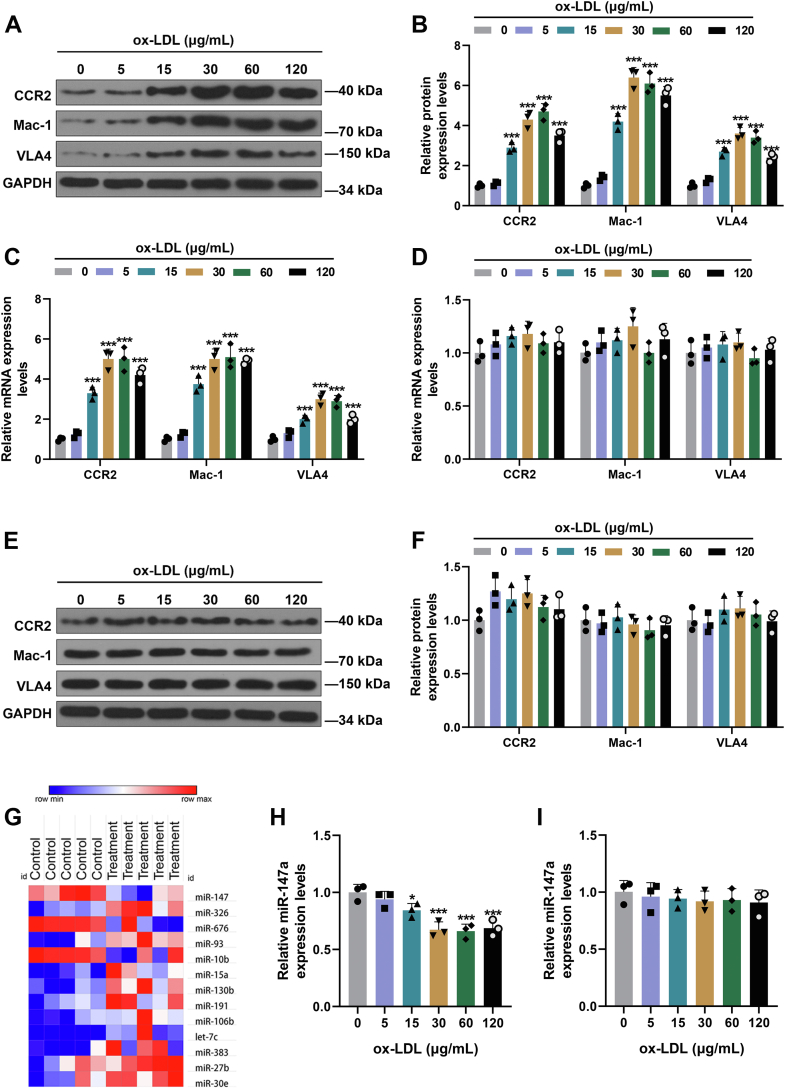
**The supernatants of ox-LDL-treated HUVECs increased expressions of mononuclear chemotactic adhesion receptors and decreased that of miR-147a in THP-1 cells.***A*–*C*, the expressions of mononuclear chemotactic adhesion receptors (CCR2, Mac-1, and VLA4) were detected by Western blot and qRT–PCR in THP-1 cells, after treatment with supernatants of ox-LDL-treated HUVECs. *D*–*F*, the expressions of mononuclear chemotactic adhesion receptors (CCR2, Mac-1, and VLA4) were detected by Western blot and qRT–PCR in THP-1 cells, after treatment with ox-LDL. *G*, validation of a subset of miRNA differentially expressed between LPS/interferon-γ-activated mouse macrophages and control group by R language software. *Blue color* represents downregulated genes, and *red color* represents upregulated genes. *H* and *I*, miR-147a level was detected in THP-1 cells treated with supernatants of ox-LDL-treated HUVECs (*H*) or ox-LDL (*I*). GAPDH was served as the internal control. ∗∗∗*p* < 0.001 *versus* 0. The data were expressed as mean ± SD. The one-way ANOVA was used to compare multiple groups, followed by post hoc Dunnett’s or Tukey’s test. CCR2, CC chemokine receptor 2; HUVEC, human umbilical vein endothelial cell; LPS, lipopolysaccharide; Mac-1, integrin αMβ2; Ox-LDL, oxidized low-density lipoprotein; qRT–PCR, quantitative RT–PCR; VLA4, very late antigen 4.

After incubation with cultural supernatants of HUVECs treated with ox-LDL (15–120 μg/ml) for 24 h, the expressions of mononuclear chemotactic adhesion receptors (very late antigen 4 [VLA4], Mac-1, and CC chemokine receptor 2 [CCR2]) in THP-1 cells were apparently elevated (Fig. 2, *A*–*C*, *p* < 0.001). Also, when compared with 0 μg/ml ox-LDL, 5 μg/ml ox-LDL barely affected these expressions. Nevertheless, when THP-1 cells were directly treated with ox-LDL at various concentrations for 24 h, the levels of mononuclear chemotactic adhesion receptors were not dramatically affected (Fig. 2, *D*–*F*). As can be seen from Figure 2*G*, heatmap analyses revealed that 13 miRNAs critical to macrophage function during AS showed differential expression levels (three were downregulated and ten were upregulated). Among them, miR-147a exhibited the most markedly downregulated expression (three fold change). Other studies also reported that miR-147a expression is notably downregulated in peripheral blood mononuclear cells of patients with CAD (15). We therefore assumed that miR-147a may exert a critical effect on functions of macrophages during AS. Furthermore, it was observed that the cultural supernatants of HUVECs treated with ox-LDL reduced miR-147a level in THP-1 cells (Fig. 2*H*, *p* < 0.05). However, when THP-1 cells were directly treated with ox-LDL at various concentrations, miR-147a level was only slightly reduced (Fig. 2*I*).

### miR-147a attenuated the adherence of monocyte to HUVECs and the upregulation of mononuclear chemotactic adhesion receptors in THP-1 cells induced by supernatants of ox-LDL-treated HUVECs

**Figure 3:**
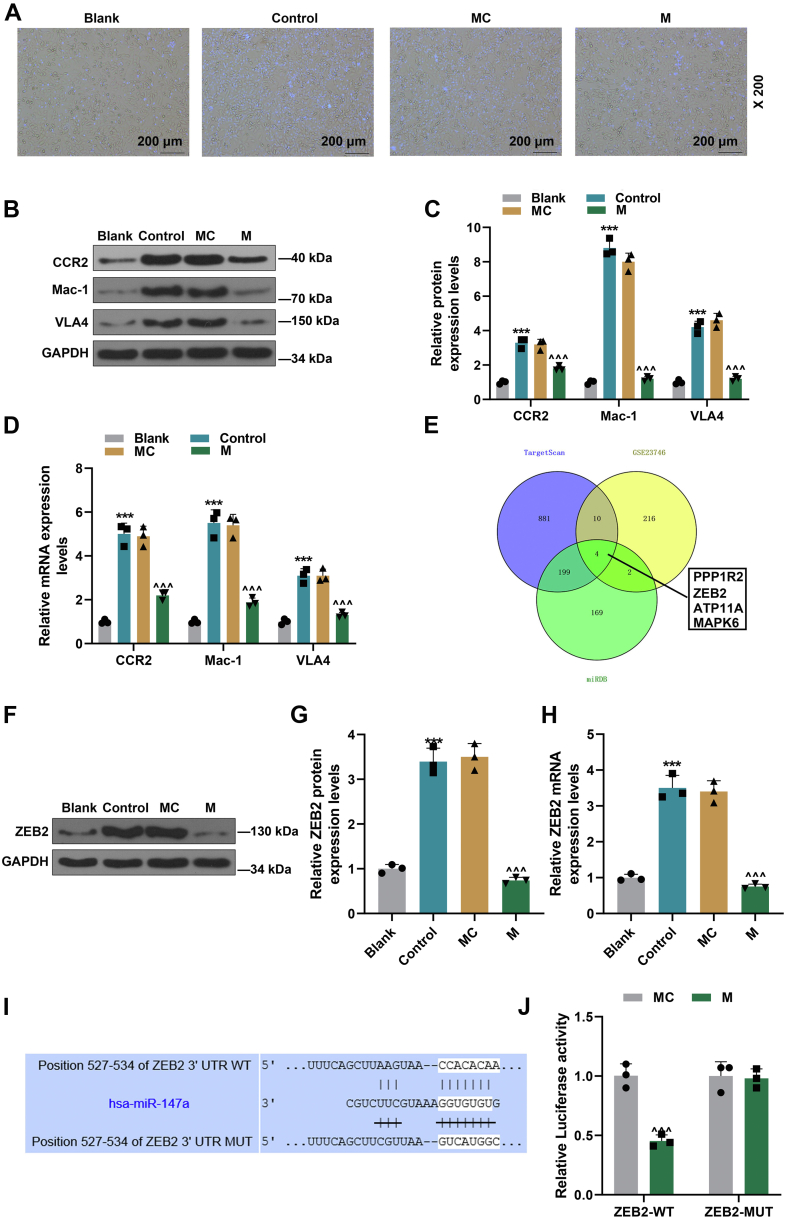
**miR-147a attenuated monocyte adherence to HUVECs and the upregulation of mononuclear chemotactic adhesion receptors in THP-1 cells induced by supernatants of ox-LDL-treated HUVECs.***A*, miR-147a attenuated monocyte adherence to HUVECs treated with ox-LDL. *B*–*D*, miR-147a attenuated the upregulation of mononuclear chemotactic adhesion receptors (CCR2, Mac-1, and VLA4) in THP-1 cells induced by supernatants of ox-LDL-treated HUVECs. *E*, Venn diagram of genes regulated by miR-147a. *F*–*H*, the expression of ZEB2 in THP-1 cells was detected by Western blot and qRT–PCR. *I* and *J*, the binding relationship between miR-147a and ZEB2 was predicted by TargetScan and verified by dual-luciferase reporter assay. GAPDH was served as the internal control. ∗∗∗*p* < 0.001 *versus* blank; ^∧∧∧^*p* < *versus* MC. The data were expressed as mean ± SD. Two groups of data were compared using an independent samples *t* test. The one-way ANOVA was used to compare multiple groups, followed by post hoc Tukey’s test. CCR2, CC chemokine receptor 2; HUVEC, human umbilical vein endothelial cell; M, miR-147a mimic; Mac-1, integrin αMβ2MC; MC, mimic control; ox-LDL, oxidized low-density lipoprotein; qRT–PCR, quantitative RT–PCR; VLA4, very late antigen 4; ZEB2, zinc finger E-box binding homeobox 2.

From Figure 3*A*, when compared with blank group, the adherence of monocyte to HUVECs was induced in control group, whereas such adherence of monocyte to HUVECs was inhibited in mimic (M) group relative to that in mimic control (MC) group. Furthermore, based on Figure 3, *B*–*D*, the levels of mononuclear chemotactic adhesion receptors (VLA4, Mac-1, and CCR2) in THP-1 cells were augmented in control group as contrasted with those in blank group (*p* < 0.001), but these levels were diminished in M group as compared with MC group (*p* < 0.001). The expressions of these mononuclear chemotactic adhesion receptors are vital for the adhesion of monocytes to HUVECs. Therefore, we speculated that the decrease of HUVEC monocyte adherence resulted from the downregulated mononuclear chemotactic adhesion receptors.

### miR-147a attenuated monocyte adherence to HUVECs and the upregulation of mononuclear chemotactic adhesion receptors in THP-1 cells induced by supernatants of ox-LDL-treated HUVECs through directly downregulating ZEB2

To identify the downstream gene mRNA regulated by the crucial miRNA, miR-147a, Venny analysis with Gene Expression Omnibus (GEO) (GSE23746), TargetScan, and miRDB was performed to screen the critical genes regulating monocyte function. In accordance with Figure 3*E*, four genes, including PPP1R2, ZEB2, ATP11A, and MAPK6, were singled out. Among them, previous studies demonstrated that ZEB2 exerts considerable influences on cancer cells, cancer stem cells, and nervous system development, which is a risk factor for vascular diseases such as AS (9, 10, 11). Hence, quantitative RT–PCR (qRT–PCR) and Western blot were conducted to further detect ZEB2 expression. The results indicated that the level of ZEB2 in THP-1 cells was higher in control group than in blank group (Fig. 3, *F*–*H*, *p* < 0.001), whereas that was lower in M group than in MC group (Fig. 3, *F*–*H*, *p* < 0.001). Moreover, the binding relationship between miR-147a and ZEB2 was verified by bioinformatics analysis (Fig. 3*I*) and dual-luciferase reporter assay (Fig. 3*J*). Thus, the aforementioned results proved that miR-147a suppressed the rise of ZEB2 expression *via* binding its 3′-UTR in THP-1 cells induced by cultural supernatants of ox-LDL-treated HUVECs.

**Figure 4:**
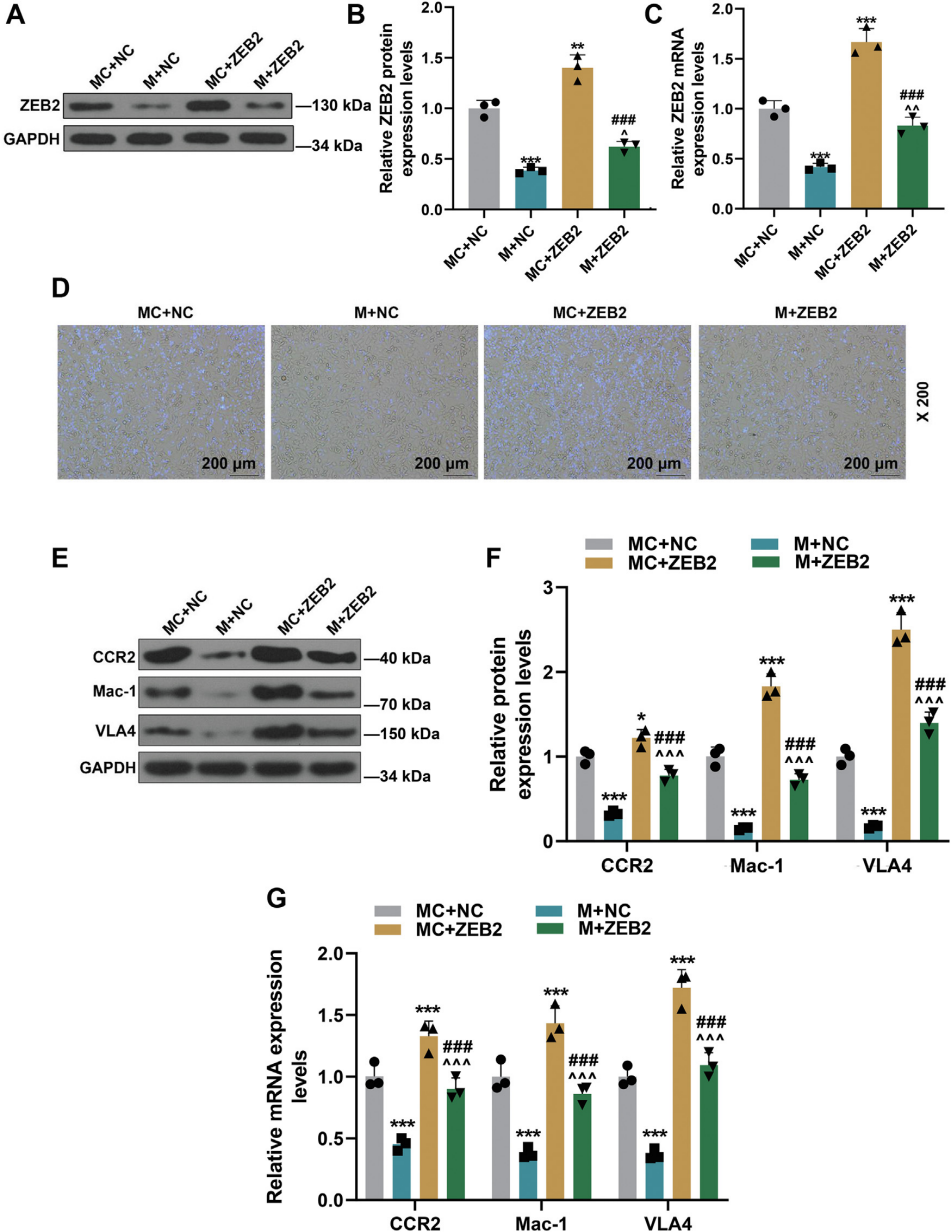
**miR-147a attenuated monocyte adherence to HUVECs and the upregulation of mononuclear chemotactic adhesion receptors in THP-1 cells induced by supernatants of ox-LDL-treated HUVECs through directly downregulating ZEB2 level.***A*–*C*, the expression of ZEB2 in THP-1 cells was detected by Western blot and qRT–PCR. *D*, ZEB2 counteracted the effect of miR-147a mimic on monocyte adherence to HUVECs. *E*–*G*, ZEB2 offset the effect of miR-147a mimic on the expressions of mononuclear chemotactic adhesion receptors (CCR2, Mac-1, and VLA4) in THP-1 cells induced by supernatants of ox-LDL-treated HUVECs. GAPDH was served as the internal control. ∗*p* < 0.05, ∗∗∗*p* < 0.001 *versus* MC + NC; ^∧∧∧^*p* < *versus* M + NC; ^###^*p* < 0.001 *versus* MC + ZEB2. NC, negative control (empty plasmid vector). The data were expressed as mean ± SD. The one-way ANOVA was used to compare multiple groups, followed by post hoc Tukey’s test. CCR2, CC chemokine receptor 2; HUVEC, human umbilical vein endothelial cell; M, miR-147a mimic; Mac-1, integrin αMβ2; MC, mimic control; ox-LDL, oxidized low-density lipoprotein; qRT–PCR, quantitative RT–PCR; VLA4, very late antigen 4; ZEB2, zinc finger E-box binding homeobox 2.

As depicted in Figure 4, *A*–*C*, ZEB2 was obviously upregulated in MC + ZEB2 group compared with that in MC + negative control (NC) group (*p* < 0.01). Relative to MC + ZEB2 group, M + ZEB2 group exhibited downregulation of ZEB2 (*p* < 0.01). In the light of Figure 4*D*, when compared with MC + NC group, HUVEC monocyte adherence was reduced in M + NC group but was increased in MC + ZEB2 group; relative to MC + ZEB2 group, HUVEC monocyte adherence was reduced in M + ZEB2 group; moreover, the increment of HUVEC monocyte adherence was the most notable in MC + ZEB2 group. The aforementioned data combined with Figure 4, *E*–*G* confirmed that through directly downregulating ZEB2 level, miR-147a inhibited HUVEC monocyte adherence and remarkably suppressed the expressions of mononuclear chemotactic adhesion receptors in THP-1 cells (*p* < 0.001).

### miR-147a influenced M1 and M2 macrophage polarization from THP-1 cells and the roles of their supernatants (THP-1 cells) in HUVEC apoptosis

**Figure 5:**
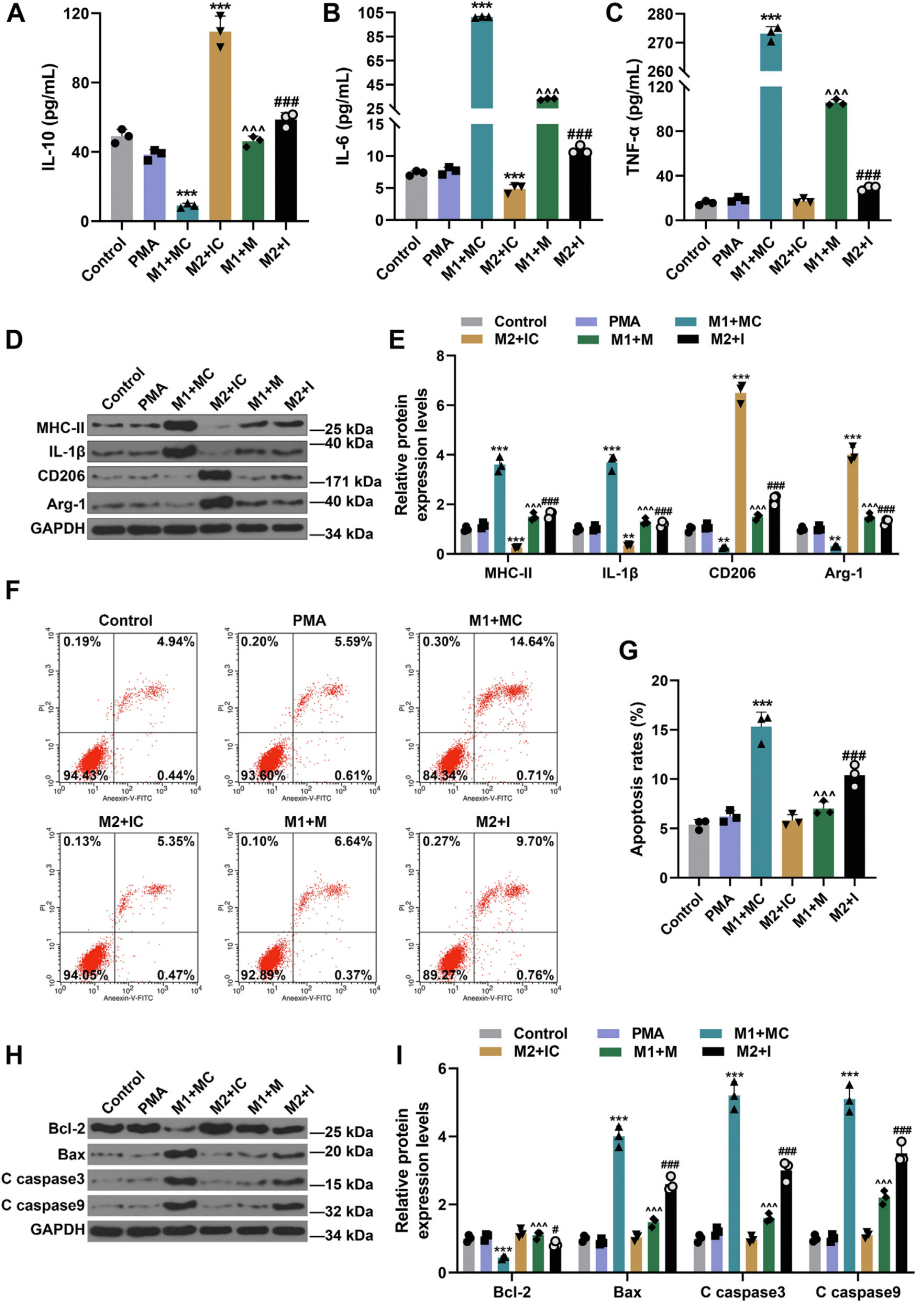
**miR-147a influenced M1 and M2 macrophage polarization from THP-1 cells and the roles of THP-1 cell supernatants in HUVEC apoptosis.***A*–*C*, the levels of M2 macrophage marker molecules (IL-10) and M1 macrophage marker molecules (IL-6 and TNF-α) in their corresponding supernatants were detected by ELISA. *D* and *E*, Western blot was applied to detect M1 macrophage marker molecules (MHC-II and IL-1β) and M2 macrophage marker molecules (CD206 and Arg-1) in M1 and M2 macrophages. *F*–*I*, apoptosis and apoptosis-related proteins (Bcl-2, Bax, C caspase 3, and C caspase 9) of HUVECs were detected by flow cytometry assay and Western blot, respectively. ∗∗*p* < 0.01, ∗∗∗*p* <0.001 *versus* PMA; ^∧∧∧^*p* < *versus* M1 + MC; ^#^*p* <0.05, ^###^*p* < 0.001 *versus* M2 + IC. PMA for induced adherent macrophages (M0 type). The data were expressed as mean ± SD. The one-way ANOVA was used to compare multiple groups, followed by post hoc Tukey’s test. HUVEC, human umbilical vein endothelial cell; IC, inhibitor control; IL, interleukin; MHC-II, major histocompatibility complex II; PMA, phorbol-12-myristate-13-acetate.

To identify the effect of miR-147a on THP-1 cells, we detected M2 macrophage marker molecule (interleukin 10 [IL-10]) and M1 macrophage marker molecules (IL-6 and tumor necrosis factor alpha [TNF-α]) in their corresponding supernatants by ELISA. Also, M1 macrophage marker molecules (major histocompatibility complex [MHC]-II and IL-1β) and M2 macrophage marker molecules (CD206 and Arg-1) in M1 and M2 macrophages were further determined by Western blot. According to the results, the levels of M2 macrophage markers (IL-10, CD206, and Arg-1) were elevated and those of M1 macrophage markers (IL-6, MHC-II, and IL-1β) except for TNF-α were dwindled in M2 + IC group (Fig. 5, *A*–*E*, *p* < 0.001), whereas the levels of M2 macrophage markers (IL-10, CD206, and Arg-1) were decreased and those of M1 macrophage markers (IL-6, TNF-α, MHC-II, and IL-1β) were increased in M1 + MC group (Fig. 5, *A*–*E*, *p* < 0.01) compared with those in phorbol-12-myristate-13-acetate (PMA) group. However, regarding these markers, their changes in M1 + M group/M2 + I group were opposite when compared with those in M1 + MC group/M2 + IC group, respectively (Fig. 5, *A*–*E*, *p* < 0.001). The results confirmed that THP-1 cells differentiated into M1 macrophage and M2 macrophage were restrained by miR-147a M and miR-147a I, respectively.

After culture with cultural supernatants of THP-1 cells subjected to miR-147a M/I transfection or/and differential induction, apoptosis rate and apoptosis-related molecules were detected by flow cytometry and Western blot assays, respectively. The results showed that the apoptosis rate was higher in M1 + MC group than in PMA group (Fig. 5, *F* and *G*, *p* < 0.001). But in contrast to M1 + MC group, the apoptosis rate was reduced in M1 + M group (Fig. 5, *F* and *G*, *p* < 0.001). Compared with that in M2 + IC group, the apoptosis rate was increased in M2 + I group (Fig. 5, *F* and *G*, *p* < 0.001). The level of Bcl-2 was decreased and those of Bax, C caspase 3, and C caspase 9 were increased in M1 + MC group in contrast with those in PMA group (Fig. 5, *H* and *I*, *p* < 0.001), whereas the changes in M1 + MC group were partially offset in M1 + M group (Fig. 5, *H* and *I*, *p* < 0.001). As compared with those in M2 + IC group, the level of Bcl-2 was reduced and those of Bax, C caspase 3, and C caspase 9 were augmented in M2 + I group (Fig. 5, *H* and *I*, *p* < 0.05). The results proved that miR-147a mimic repressed differentiation of M1 macrophage from THP-1 cells, which restrained HUVEC apoptosis; miR-147a inhibitor repressed differentiation of M2 macrophage from THP-1 cells, which promoted HUVEC apoptosis.

### miR-147a upregulation in macrophages reduced atherosclerotic plaques through inhibiting ZEB2 expression in AS mice

**Figure 6:**
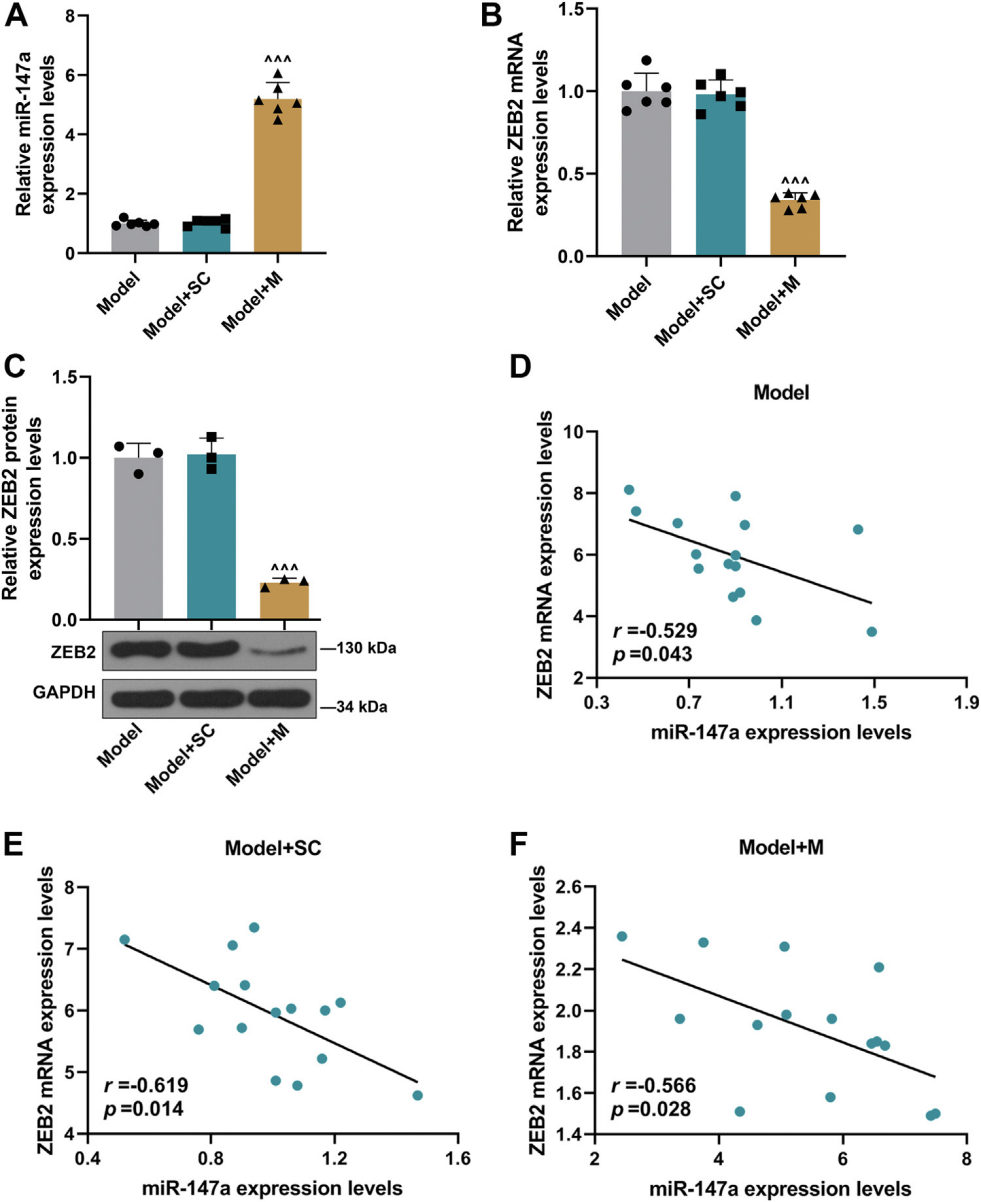
**miR-147a upregulation in macrophages suppressed ZEB2 expression in atherosclerotic plaques of AS mice.***A*, qRT–PCR indicated the level of miR-147a in model, model + SC, and model + M groups. *B* and *C*, the level of ZEB2 in model, model + SC, and model + M groups was indicated by qRT–PCR and Western blot. *D–F*, the correlation between miR-147a and its downstream target ZEB2 in model, model + SC, and model + M groups was analyzed by Pearson’s correlation. U6 or GAPDH was served as the internal control. ^∧∧∧^*p* < 0.001 *versus* model + SC. Model, AS model; model + SC, AS model + agomir control; model + M, AS model + miR-147a agomir. The data were expressed as mean ± SD. The one-way ANOVA was used to compare multiple groups, followed by post hoc Tukey’s test. AS, atherosclerosis; M, miR-147a mimic; qRT–PCR, quantitative RT–PCR; ZEB2, zinc finger E-box binding homeobox.

It was found that in atherosclerotic plaques of AS mice, when compared with model + agomir control (SC) group, model + M group presented upregulation of miR-147a (Fig. 6*A*, *p* < 0.001) but downregulation of ZEB2 (Fig. 6, *B* and *C*, *p* < 0.001). Moreover, miR-147a level had an inverse correlation with ZEB2 level in model, model + SC, and model + M groups (Fig. 6, *D*–*F*, *p* < 0.05). The data reflected that miR-147a upregulation inhibited ZEB2 expression in atherosclerotic plaques of AS mice.

**Figure 7:**
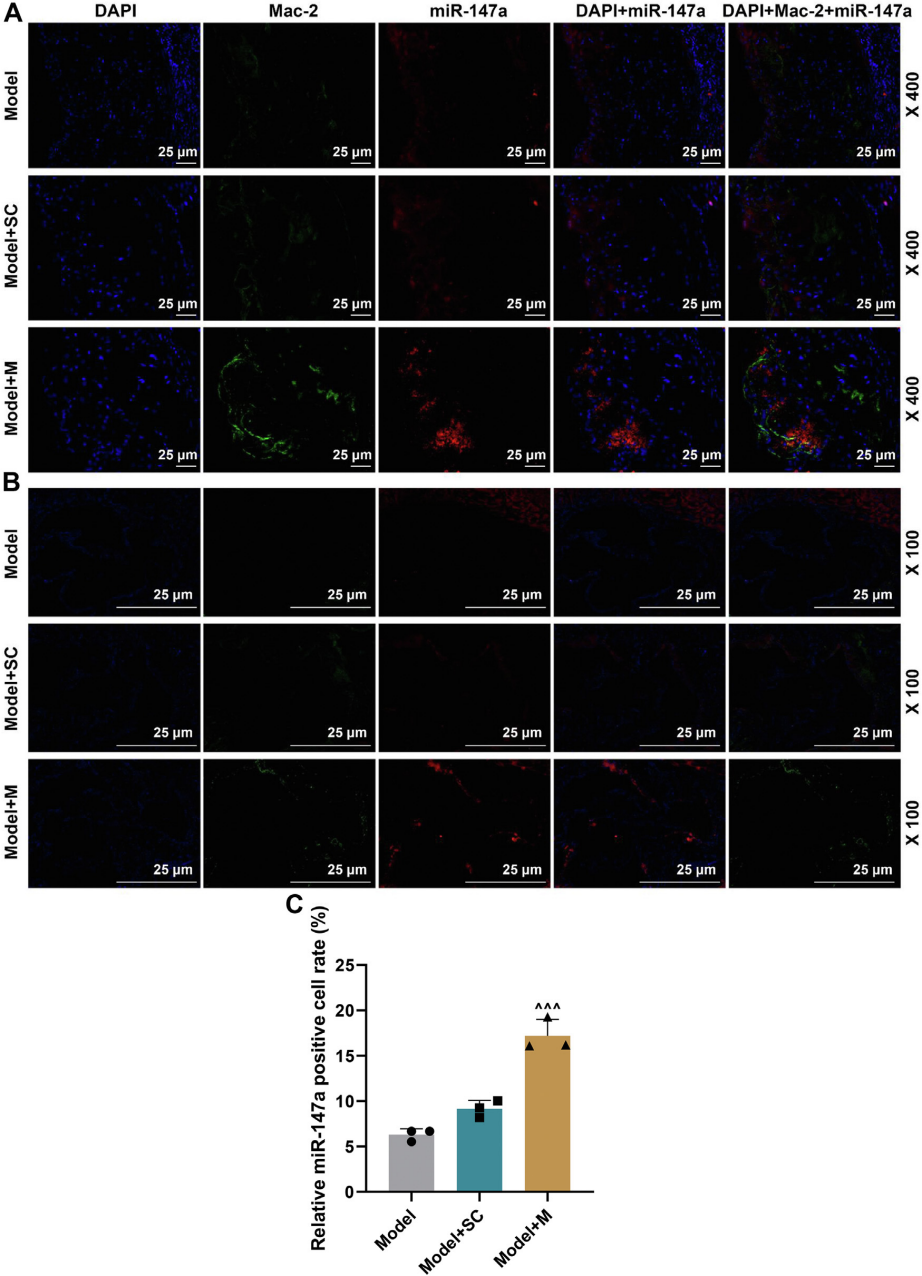
**Cellular localization of miR-147a in macrophagesof atherosclerotic plaques in AS mice.***A* and *B*, *in situ* hybridization (ISH) and immunofluorescence were performed on mouse aortic arch of model, model + SC, and model + M groups for cellular localization of miR-147a and Mac-2. *Blue* is 4′,6-diamidino-2-phenylindole, *green* is an antibody against Mac-2, and *red* is a probe exclusively against miR-147a. 100× and 400× magnification. *C*, quantification of positive rate of miR-147a^+^ cells. ^∧∧∧^*p* < 0.001 *versus* model + SC. Model, AS model; model + SC, AS model + agomir control; and model + M, AS model + miR-147a agomir. The data were expressed as mean ± SD. The one-way ANOVA was used to compare multiple groups, followed by post hoc Tukey’s test. AS, atherosclerosis; M, miR-147a mimic.

**Figure 8:**
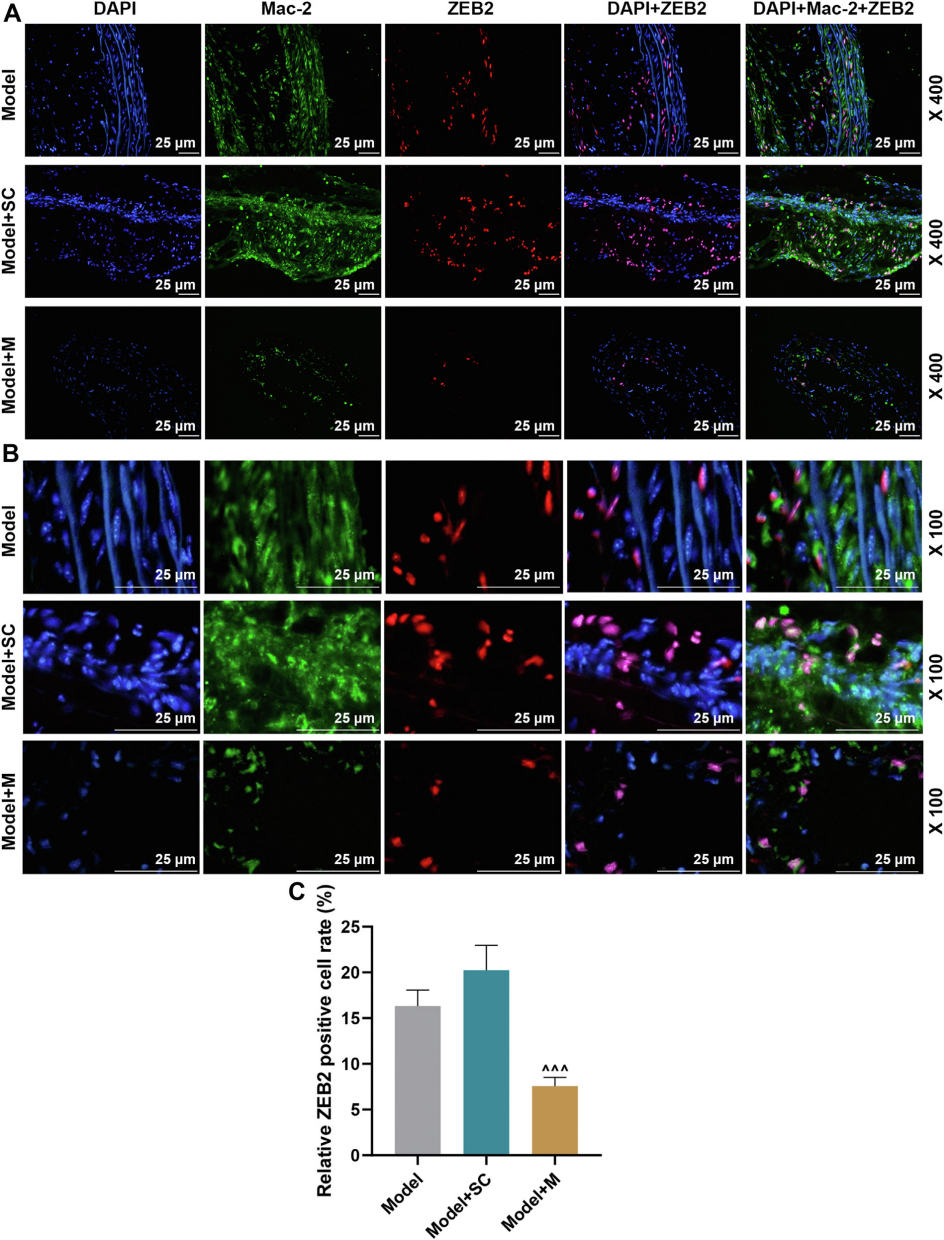
**Cellular localization of ZEB2 in macrophagesof atherosclerotic plaques in AS mice.***A* and *B*, immunofluorescence was performed on mouse aortic arch of model, model + SC, and model + M groups for cellular localization of ZEB2 and Mac-2. *Blue* is 4′,6-diamidino-2-phenylindole, *green* is an antibody against Mac-2, and *red* is an antibody against ZEB2. 100× and 400× magnification. *C*, quantification of positive rate of ZEB2^+^ cells. ^∧∧∧^*p* < 0.001 *versus* model + SC. Model, AS model; model + SC, AS model + agomir control; model + M, AS model + miR-147a agomir. The data were expressed as mean ± SD. The one-way ANOVA was used to compare multiple groups, followed by post hoc Tukey’s test. AS, atherosclerosis; M, miR-147a mimic; ZEB2, zinc finger E-box binding homeobox 2.

In line with Figure 7, *A* and *B*, *in situ* hybridization (ISH) and immunofluorescence assays showed the cellular localization of miR-147a and Mac-2 in atherosclerotic plaques of AS mice in model, model + SC, and model + M groups. Compared with the model + SC group, the positive rate of miR-147a^+^ cells was markedly increased in model + M group (Fig. 7*C*, *p* < 0.001). In Figure 8, *A* and *B*, immunofluorescence assay showed the cellular localization of ZEB2 and Mac-2 in atherosclerotic plaques of AS mice in model, model + SC, and model + M groups. In Figure 8*C*, in contrast to the model + SC group, the positive rate of ZEB2^+^ cells was signally decreased in model + M group (Fig. 8*C*, *p* < 0.001). Substantial β-galactoside-binding members in the lectin family, galectin-3 (also known as Mac-2), are expressed in macrophages, which drives inflammation, fibroblast proliferation, and collagen production (16). Therefore, the results indicated that miR-147a and ZEB2 localized at macrophages of atherosclerotic plaques in AS mice.

**Figure 9:**
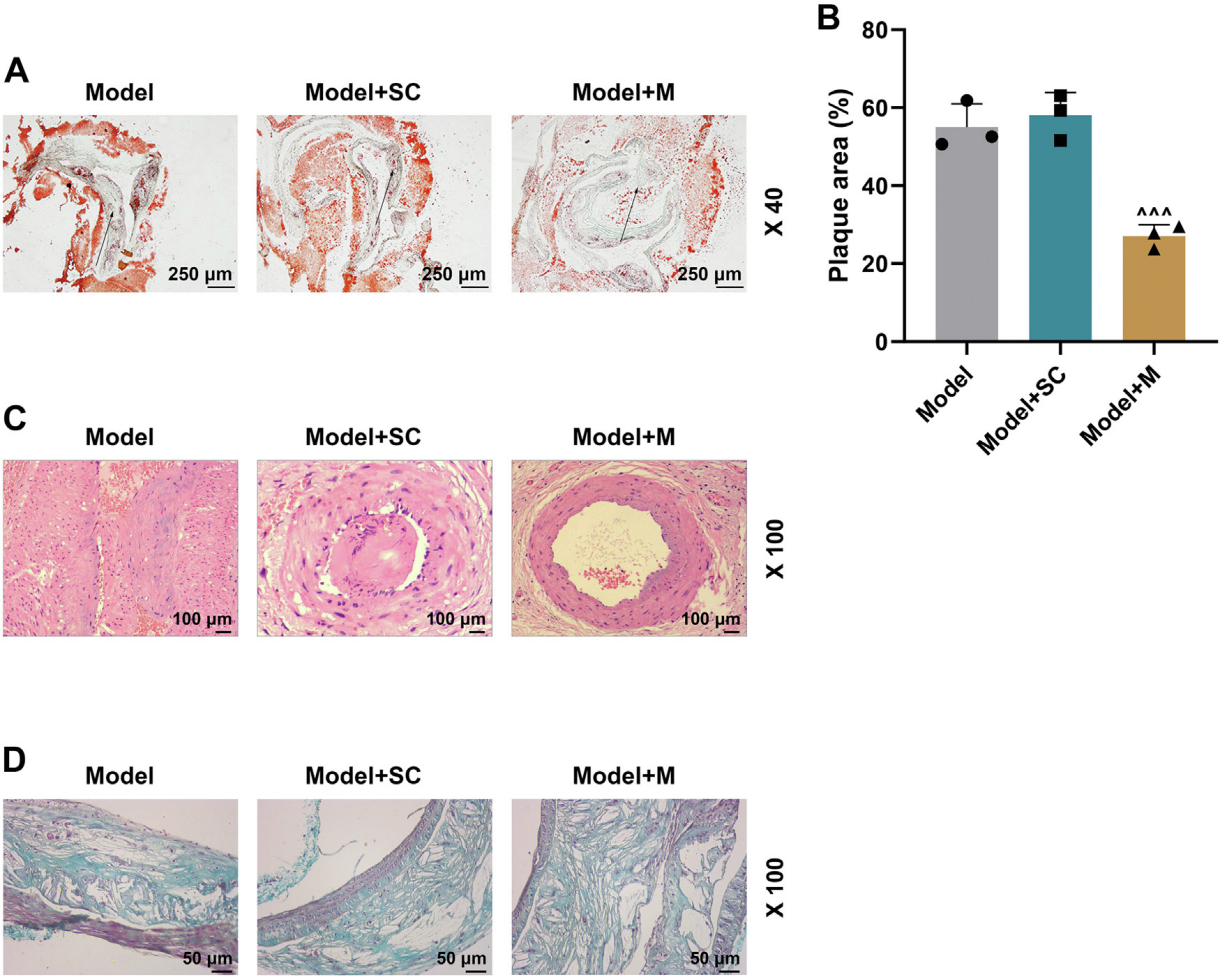
**miR-147a upregulation reduced atherosclerotic plaques in AS mice.***A*, Oil Red O staining was performed for determining atherosclerotic plaques in aortic arch of AS mice. *Black arrows*: large amounts of foam and lipid accumulation in the blood vessels. *B*, quantification of lesion size. 40× magnification. *C*, effects of miR-147a mimic on atherosclerotic plaques stained with HE. *D*, the effects of miR-147a mimic on collagen content in the atherosclerotic lesions stained with Masson staining. ^∧∧∧^*p* < 0.001 *versus* model + SC. Model, AS model; model + SC, AS model + agomir control; model + M, AS model + miR-147a agomir. The data were expressed as mean ± SD. The one-way ANOVA was used to compare multiple groups, followed by post hoc Tukey’s test. AS, atherosclerosis; HE, hematoxylin–eosin.

From Figure 9, *A*–*D*, massive lipid disposition, foam cell formation, atherosclerotic plaques, and collagen content were found in model and model + SC groups. However, by contrast, such accumulations in model + M group were reduced (*p* < 0.001). The results proved that miR-147a suppressed atheromatous plaque lesions in AS mice.

### miR-147a upregulation suppressed expressions of mononuclear chemotactic adhesion receptors and M1 polarization but promoted M2 polarization in AS mice

**Figure 10:**
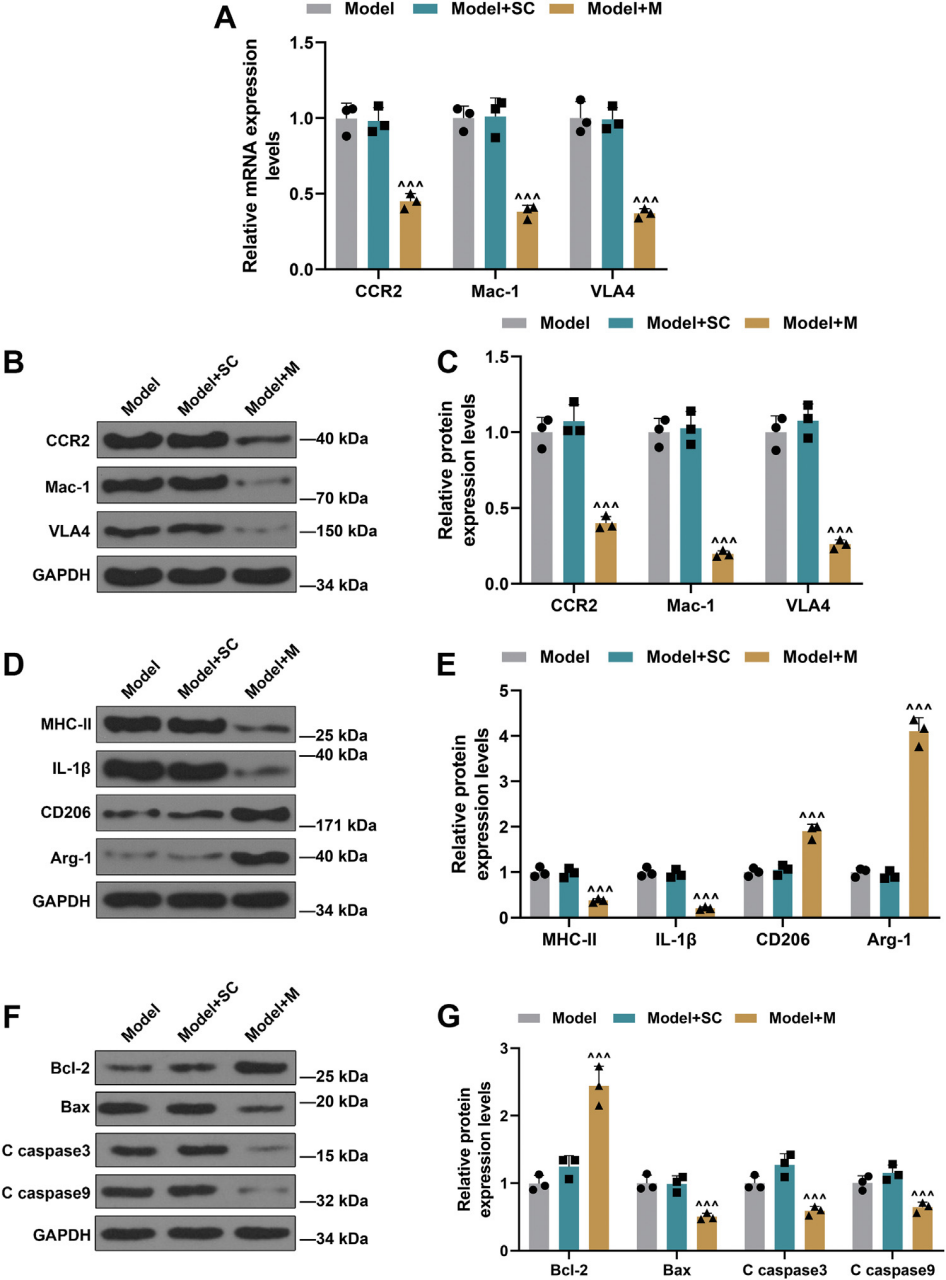
**miR-147a upregulation suppressed expressions of mononuclear chemotactic adhesion receptors and M1polarization but promoted M2 polarization in AS mice.***A*–*C*, the expressions of mononuclear chemotactic adhesion receptors (CCR2, Mac-1, and VLA4) were detected by qRT–PCR and Western blot. *D* and *E*, Western blot was applied to detect M1 macrophage marker molecules (MHC-II and IL-1β) and M2 macrophage marker molecules (CD206 and Arg-1) in atherosclerotic plaques of AS mice. *F* and *G*, apoptosis-related proteins (Bcl-2, Bax, C caspase 3, and C caspase 9) were detected by Western blot. GAPDH was served as the internal control. ^∧∧∧^*p* < 0.001 *versus* model + SC. Model, AS model; model + SC, AS model + agomir control; model + M, AS model + miR-147a agomir. The data were expressed as mean ± SD. The one-way ANOVA was used to compare multiple groups, followed by post hoc Tukey’s test. AS, atherosclerosis; CCR2, CC chemokine receptor 2; IL-1β, interleukin 1β; Mac-1, integrin αMβ2; MHC-II, major histocompatibility complex II; qRT–PCR, quantitative RT–PCR; VLA4β1, integrin very late antigen 4.

The expressions of mononuclear chemotactic adhesion receptors (VLA4, Mac-1, and CCR2) were suppressed in model + M group compared with those in model + SC group (Fig. 10, *A*–*C*, *p* < 0.001), indicating miR-147a upregulation impeded monocyte adherence to vascular endothelial cells. In addition, the expressions of M1 macrophage marker molecules (MHC-II and IL-1β) were suppressed in model + M group, whereas those of M2 macrophage marker molecules (CD206 and Arg-1) were promoted compared with those in model + SC group (Fig. 10, *D* and *E*, *p* < 0.001). The comparative results verified that miR-147a upregulation suppressed M1 macrophage differentiation but promoted M2 macrophage differentiation in AS mice. The levels of Bax, C caspase 3, and C caspase 9 were prominently decreased but that of Bcl-2 was significantly increased in model + M group, as compared with those in model + SC group (Fig. 10, *F* and *G*, *p* < 0.001).

## Discussion

Ox-LDL is a widely acknowledged factor in the formation of AS (17). In this study, we found that miR-147a attenuated monocyte adherence to ox-LDL-injured HUVECs and the upregulation of mononuclear chemotactic adhesion receptors through directly downregulating ZEB2 expression in THP-1 cells, which were induced by supernatants of ox-LDL-injured HUVECs. Besides, miR-147a targeted ZEB2 to influence the formation of atherosclerotic plaques through regulating M1/M2 polarization and macrophage adhesion in AS mice *in vivo*.

sICAM-1 is released from cell-surface ICAM-1 by proteolytic cleavage in response to inflammatory cytokines or endothelial injury. Endothelial cells acquire a proinflammatory phenotype and start expressing different proinflammatory molecules, such as MCP-1 (also known as chemokine [C-C motif] ligand 2), ICAM-1, and VCAM-1, all of which attract monocytes to the activated endothelium (18). MCP-1 is a potent chemotactic factor for monocyte that functions through its receptor CCR2 (19, 20) and induces the migration, aggregation, adhesion, and activation of monocytes to the injury site in the arterial wall, finally causing vascular injury (21). As a member of the immunoglobulin superfamily, ICAM-1 is widely present and expressed in various cells including vascular endothelial cell membrane, with its main receptor Mac-1 mainly expressed in monocytes and neutrophils (22). VCAM-1 belongs to the immunoglobulin superfamily, whose receptor VLA4 is mainly expressed in mononuclear macrophages. ICAM-1/Mac-1 and VCAM-1/VLA-4 interaction mediates the close adhesion of monocytes to vascular endothelium and further promotes the migration of monocytes to the endothelium (23). The present results suggested that ox-LDL causes injury and activation of HUVECs accompanied with high expressions of chemokines (MCP-1) and soluble adhesion molecules (sICAM-1 and sVCAM-1), which in turn promotes the expressions of mononuclear chemotactic adhesion receptors (VLA4, Mac-1, and CCR2) in THP-1 cells. All these attract THP-1 cells to the activated HUVECs. A similar report also showed that the downregulation in the contents of MCP-1, sICAM-1, and sVCAM-1 in HUVECs leads to U937-HUVEC adhesion decrease (24). We also verified that HUVECs secrete high expressions of MCP-1 and sICAM-1 and sVCAM-1 when exposed to ox-LDL, thus influencing the cascade of events in THP-1 cells and ultimately regulating miR-147a and ZEB2 expressions. However, besides VCAM-1 and ICAM-1, impaired function of human coronary artery endothelial cell and induced expression of lectin-like ox-LDL receptor (LOX)-1 also regulate monocyte adhesion and infiltration (25). Therefore, in addition to secreting MCP-1, sICAM-1, and sVCAM-2, other factors that are secreted by HUVECs may also impact the fate of THP-1 cells, miR-147a and ZEB2. Interfering with miRNA expression in the artery wall is a potential way to affect atherosclerotic plaque and cardiovascular disease development (26). In accordance with bioinformatics analysis in our study and a previous report (15), miR-147a is speculated to play a critical role in regulating macrophage function during AS. In the present study, it was explored that miR-147a level was significantly decreased in THP-1 cells after treatment with ox-LDL-cultured HUVEC supernatants but was only slightly lessened in THP-1 cells after direct treatment with ox-LDL, which reflected that miR-147a may be involved in the effect of ox-LDL-cultured HUVEC supernatants on THP-1 cells. Moreover, the present study pointed out that miR-147a mimic attenuated monocyte adherence to HUVECs and the upregulation of mononuclear chemotactic adhesion receptors in THP-1 cells induced by ox-LDL-cultured HUVEC supernatants. The aforementioned research results confirmed that miR-147a upregulation may resist monocyte adherence to HUVECs, thus reducing macrophage-derived foam cells, inhibiting the formation of lipid stripes and lipid plaques, and playing an anti-AS role.

It is known that each miRNA might have many mRNA targets, and each mRNA could also be regulated by more than one miRNA (27). Previous reports have demonstrated that ZEB2 is a risk factor for vascular diseases such as AS (9, 10, 11). Furthermore, Ma *et al.* (12) have identified three independent risk SNPs of CAD in close proximity to the ZEB2 coding region, and these SNPs possibly function in concert with the atherosclerotic arterial wall and adipose tissues, through modulation of metabolism and lipid. In addition, by bioinformatics analysis in our study, ZEB2, the predictive target gene of miR-147a, was selected for our subsequent researches. Our results also confirmed that miR-147a mimic directly inhibited ZEB2 expression through binding to its 3′-UTR. Moreover, the present results showed that ZEB2 overexpression partially weakened the inhibitory effect of miR-147a upregulation on monocyte adherence to HUVECs and the upregulation of mononuclear chemotactic adhesion receptors in THP-1 cells induced by ox-LDL-cultured HUVEC supernatants. The aforementioned research results validated that miR-147a regulated monocyte adherence to HUVECs induced by ox-LDL-cultured HUVEC supernatants through binding to ZEB2.

Macrophages are classified into two types: proinflammatory M1 and anti-inflammatory M2 macrophages (28). A study has confirmed that AS presence of M1 and M2 macrophages at the lesion site is a primary factor in plaque formation, fibrous cap rupture, and thrombosis (29). The M1 type is dominant in unstable plaque tissue but M2 type in stable plaque tissue (30). M1 type macrophages play leading roles in the early AS formation owing to its proinflammatory and tissue damage effects, whereas M2 type macrophages release anti-inflammatory factors to eliminate inflammatory response, promote the formation of plaque fibrous cap, and enhance plaque stability (31). In the present study, we unveiled that the upregulation and downregulation of miR-147a suppressed M1 type polarization and M2 type polarization of macrophages, respectively, which was reflected by the expressions of M1 markers and M2 markers. Furthermore, inflammatory factors, secreted by M1 macrophages, are important damage factors of vascular endothelium, which inevitably cause damage to vascular endothelium. The present data suggested that miR-147a upregulation inhibited the differentiation of M1 macrophage from THP-1 cells, apoptosis of HUVECs and apoptosis-related molecules (Bax, C caspase 3, and C caspase 9), whereas miR-147a downregulation repressed differentiation of M2 macrophage from THP-1 cells and promoted apoptosis of HUVECs. The aforementioned research results indicated that miR-147a could regulate M1/M2 phenotypic polarization of macrophages and modulate secretion of inflammatory factors (M1 type) and anti-inflammatory factors (M2 type), subsequently influencing the roles of M1 type and M2 type macrophages in apoptosis of HUVECs.

Our findings also revealed that miR-147a upregulation in macrophages suppressed atherosclerotic plaques through inhibiting ZEB2 expression in AS mice. miR-147a upregulation inhibited expressions of mononuclear chemotactic adhesion receptors and M1 polarization but boosted M2 polarization in AS mice. Phagocytic macrophages are actively implicated in lipid accumulation by engulfing lipid droplets, leading to foam cells (1). M1 macrophages play a leading role in early AS formation on account of its proinflammatory and tissue-damaging effects, whereas M2 macrophages release anti-inflammatory factors, eliminate inflammatory response, promote the formation of plaque fibrous cap, and enhance plaque stability (31). These aforementioned researches unraveled that the miR-147–ZEB2 axis impacted early formation and late stability of atherosclerotic plaques through regulating M1/M2 macrophage polarization and macrophage adhesion to vascular endothelial cells in AS mice. Collectively, our data demonstrated that miR-147a regulated monocyte adherence to HUVECs in THP-1 cells induced by ox-LDL-cultured HUVEC supernatants, M1/M2 phenotypic polarization, and their roles in apoptosis of HUVECs through directly downregulating ZEB2 level. Moreover, miR-147a modulated macrophages that engulf lipid-forming lipid stripes and atherosclerotic plaques, lipid stripe formation, M1/M2 phenotypic polarization, and monocyte adherence to endothelial cells through directly downregulating ZEB2 level in AS mice. It is important to highlight the limitation in this study. We used only male mice in this study to avoid the potential confounding effects of estrous cycle and sex hormones in female mice. However, sex bias and constrained generalizability of the data in females emerge.

The present study provides new mechanistic insights into a crucial part for miR-147a–ZEB2 axis in plaque formation and stabilization of AS as well as theoretical basis for the treatment of AS. The findings may be conducive to developing a novel targeting therapeutic method for AS so as to reduce AS-related mortality.

## Experimental procedures

### Ethics statement

All animal experiments were performed in compliance with the guidelines of the China Council on Animal Care and Use. This study was recommended by the Committee of Experimental Animals of The Affiliated Hospital of Inner Mongolia Medical University (approval number: NMGYKDX20200415018). Every effort was made to minimize pain and discomfort to the animals. The animal experiments were conducted in The Affiliated Hospital of Inner Mongolia Medical University.

### Bioinformatics analysis and dual-luciferase reporter assay

Raw data (GSE33453 and GSE87718) from the GEO database (https://www.ncbi.nlm.nih.gov/geo/) were first analyzed to compare the miRNA expression profiles in macrophages using GEO2R (https://www.ncbi.nlm.nih.gov/geo/geo2r/), an online interactive analysis tool for the GEO database on the basis of R language. Heat map analyses were performed using R language to screen overlapping differential miRNAs, which contributed to identifying the critical miRNAs in macrophage function during AS.

To identify the downstream gene mRNA regulated by the critical miRNA, miR-147a, Venny analysis with GEO (GSE23746), TargetScan, and miRDB were adopted to screen the critical genes regulating monocyte function.

The binding relationship between miR-147a and ZEB2 was predicted by TargetScan. To verify their relationship, Western blot and dual-luciferase reporter assay were conducted. Western blot was performed to detect the expression of ZEB2 in THP-1 cells after transfection with miR-147a M or miR-147a MC. To carry out dual-luciferase reporter assay, the pmirGLO vector (E1330; Promega) containing WT or mutant (MUT) ZEB2 3′-UTR was constructed. Human embryonic kidney 293 cells (catalog no.: CRL-157; American Type Culture Collection [ATCC]) were seeded in a 24-well plate overnight and were then cotransfected with ZEB2-WT vector/ZEB2-MUT vector and miR-147a M/MC. After 24 h of transfection, the cells were lysed by dual-luciferase reporter system (catalog no.: E1910; Promega) for luciferase activity detection.

### Cell culture

HUVECs were purchased from Cell Bank of the Chinese Academic of Sciences. Cryopreserved HUVECs were thawed and grown in Dulbecco's modified Eagle's medium (catalog no.: 11995; Solarbio), supplemented with 10% fetal bovine serum (catalog no.: 11011-8611; Sijiqing) and 1% penicillin–streptomycin liquid (catalog no.: P1400; Solarbio) in a humidified incubator at 37 °C with 5% CO_2_. The human monocyte cell line, THP-1 (ATCC; catalog no.: TIB-202), was obtained from the ATCC. THP-1 cells were maintained in suspension in RPMI1640 (catalog no.: 11875; Solarbio) medium containing 10% fetal bovine serum and 1% penicillin–streptomycin liquid in a humidified incubator at 37 °C with 5% CO_2_. These cell cultures were passaged 2 to 3 times per week (1:21:3 split).

### Cell grouping and treatment

An *in vitro* Ox-LDL-induced HUVEC injury model was established. Human ox-LDL (catalog no.: YB-002) was purchased from Yiyuan Biotechnologies. miR-147a M, miR-147a I, and their corresponding controls [MC], inhibitor control [IC]), as well as ZEB2 overexpressing plasmid and its NC (empty pcDNA3.1 vector) (catalog no.: V79020; ThermoFisher) were ordered from RiboBio Co, Ltd.

The HUVECs were incubated with ox-LDL at different final concentrations (0–120 μg/ml) for 24 h. For adhesion assay of monocytic adhesion on endothelial cells, the media were discarded 24 h after ox-LDL treatment, and the HUVECs were washed twice by PBS. Then the cells were further cultured with new media for 24 h. After that, the adhesion assay was performed. In addition, for quantifying the expressions of adhesion molecules (MCP-1, sICAM-1, and sVCAM-1), the media were discarded 24 h after ox-LDL treatment, and the HUVECs were washed by PBS twice. Then the cells were further incubated with new media for 24 h. Later, the cultural supernatants were collected for detecting the expressions of adhesion molecules and also used as conditioned medium for THP-1 cells. After THP-1 cells were incubated with either conditioned medium or ox-LDL for 24 h, the expressions of mononuclear chemotactic adhesion receptors (VLA4, Mac-1, and CCR2) and miR-147a in THP-1 cells were measured by Western blot and qRT–PCR, and miR-147a expression was determined *via* qRT–PCR.

To determine the function of miR-147a on THP-1 cells affected by the excreted factors (cultural supernatants) from ox-LDL-injured HUVECs, THP-1 cells were divided into blank, control, MC, and M groups. For blank group, THP-1 cells without transfection were incubated with cultural supernatants (from untreated HUVECs) for 24 h. For control group, THP-1 cells without transfection were cultivated with cultural supernatants (from 30 μg/ml ox-LDL-treated HUVECs) for 24 h. For MC or M groups, after transfection with miR-147a MC or M for 24 h, THP-1 cells were further cultured with cultural supernatants (from 30 μg/ml ox-LDL-treated HUVECs) for 24 h. Then the adhesion assay was performed, and the expressions of mononuclear chemotactic adhesion receptors (VLA4, Mac-1, and CCR2) and ZEB2 in THP-1 cells were detected by Western blot and qRT–PCR.

To reveal the interaction of ZEB2 and miR-147a on THP-1 cells affected by the excreted factors (cultural supernatants) from ox-LDL-injured HUVECs, THP-1 cells were divided into MC + NC, M + NC, MC + ZEB2, and M + ZEB2 groups. In MC + NC group, THP-1 cells were transfected with MC and empty vector for 24 h. In M + NC group, THP-1 cells were transfected with miR-147a M and empty vector for 24 h. In MC + ZEB2 group, THP-1 cells were transfected with MC and ZEB2 overexpression vector for 24 h. In M + ZEB2 group, THP-1 cells were transfected with miR-147a M and ZEB2 overexpression vector for 24 h. After transfection, THP-1 cells in each group were further incubated with cultural supernatants (from 30 μg/ml ox-LDL-treated HUVECs) for 24 h. Then the adhesion assay was conducted, and the expressions of mononuclear chemotactic adhesion receptors (VLA4, Mac-1, and CCR2) in THP-1 cells were detected by Western blot and qRT–PCR.

Under certain conditions, the two types of macrophages can be transformed into each other, and the proportion of M1 type and M2 type macrophages can significantly affect the stability of plaque. Inflammatory factors, secreted by M1 macrophages, are important damage factors of vascular endothelium and thus may cause damage to vascular endothelium. As a consequence, to further explore the function of miR-147a on the effect of excreted factors (cultural supernatants from macrophages) toward HUVECs, cultural supernatants from monocyte-derived macrophages transfected with miR-147a M or miR-147a I were collected and then used to stimulate HUVECs. First, THP-1 cells were transfected with miR-147a M, MC, miR-147a I, or IC for 24 h. Second, transfected or untransfected THP-1 cells were incubated with PMA (100 ng/ml) for 48 h to induce adherent macrophages (M0 type). As indicated, the adherent THP-1-derived macrophages transfected with miR-147a M or MC were exposed to granulocyte–macrophage colony-stimulating factor (10 ng/ml) for 24 h to induce M1 polarization, whereas adherent macrophages transfected with miR-147a I or IC were exposed to M-CSF (10 ng/ml) for 24 h to induce differentiation into M2 polarization (32). Thereafter, these cells were washed by PBS twice to remove dead or nonadherent cells and then grown in complete RPMI1640 medium. Following 24 h culture, the cultural supernatants were separately collected from M1 or M2 macrophages (transfection with miR-147a M, MC, miR-147a I, or IC) and subsequently exposed to centrifugation (2000 r/min, 20 min). The resulting supernatants were harvested as M1 + MC, M2 + IC, M1 + M, or M2 + I conditioned medium (stored at −20 °C). ELISA was applied to detect M2 macrophage marker molecules (IL-10) and M1 macrophage marker molecules (IL-6 and TNF-α) in their corresponding supernatants, respectively. Western blot was employed to determine M1 macrophage marker molecules (MHC-II and IL-1β) and M2 macrophage marker molecules (CD206; Arg-1) in M1 and M2 macrophages, respectively. Finally, after M1 and M2 macrophages were identified, HUVECs were divided into six groups: control, PMA, M1 + MC, M2 + IC, M1 + M, and M2 + I groups. HUVECs incubated with cultural supernatants from THP-1 cells (without any treatment) were set as the control group, whereas those with cultural supernatants from adherent THP-1-derived macrophages (M0 type without transfection) were perceived as the PMA group. In addition, HUVECs incubated with M1 + MC, M2 + IC, M1 + M, or M2 + I conditioned medium were set as the M1 + MC, M2 + IC, M1 + M, and M2 + I groups, respectively. Subsequently, HUVEC apoptosis rates in each group were measured by flow cytometry. Western blot was applied to detect the expressions of apoptosis-related molecules (Bcl-2, Bax, C caspase 3, and C caspase 9) in each group. Cell culture experiments along with figures were shown in Figs. S1–S3.

### Animal experimental design

A total of 45 male apolipoprotein E knockout (ApoE^−/−^) mice (C57BL/6N strain, aged 6–8 weeks, weighting 18–26 g) were purchased from Huafukang Bio-Technology Company, maintained at 20 to 30 °C with a natural light–dark cycle and randomly assigned to three groups (n = 15 each group) as follows: model (fed with a high-cholesterol diet [HCD]), model + SC (fed with HCD and injected with agomir control), and model + M (fed with HCD and injected with miR-147a agomir).

To establish the AS model in ApoE^−/−^ mice, ApoE^−/−^ mice were fed with HCD (Altromin) comprising 21% crude fat, 0.15% cholesterol, and 19.5% casein for 3 months, as previously reported (33).

### Agomir treatment *in vivo*

A chemically modified antisense RNA oligonucleotide against miR-147a and an agomir control were synthesized by RiboBio Co, Ltd. After 2 months of HCD feeding, ApoE^−/−^ mice were intravenously injected four times with miR-147a agomir or agomir control (20 nmol/20 g). The application was repeated twice a week. The aortic arch was harvested 1 week after the last injection. The necrotic core in aortic arch was identified as the part of the atherosclerotic plaque.

### Evaluation of HUVEC viability

The HUVECs were seeded into a 96-well plate at a density of 2 × 10^3^ cells/well and grown at 37 °C overnight under 5% CO_2_. The cultured cells were treated with the ox-LDL at various concentrations (0–120 μg/ml) for 24 h. Finally, the cell viability was measured with the 3-[4,5-dimethylthiazol-2-yl]-2,5 diphenyl tetrazolium bromide assay. Briefly, after treatment, 20 μl 3-[4,5-dimethylthiazol-2-yl]-2,5 diphenyl tetrazolium bromide solution (5 mg/ml, pH = 7.4, prepared with PBS; catlog no.: M8180; Solarbio) was added into each well, and the plate was further incubated for 4 h. The supernatant was discarded, 150 μl dimethyl sulfoxide (catalog no.: D8370; Solarbio) was added to each well, and the plate was oscillated for 10 min. The absorbance value of each well was measured at a wavelength of 490 nm under an enzyme-linked immunometric meter. The cell viability was calculated according to the following formula: cell viability (%) = (absorbance at 490 nm [experiment] − absorbance at 490 nm [bank])/(absorbance at 490 nm [control] − absorbance at 490 nm [bank]) × 100%. The HUVECs incubated with 0 μg/ml ox-LDL for 24 h were set as the untreated control group.

### LDH assay

HUVECs were cultured in the 96-well plate and grew to confluence of about 80%. After treatment with the ox-LDL at various concentrations (0–120 μg/ml) for 24 h, LDH activity in the culture medium was detected based on the manufacturer’s protocol of the LDH kit (catalog no.: BC0685; Solarbio).

### Adhesion assay of monocyte adhesion to endothelial cells

The determination of monocyte adhesion to the endothelial cells was conducted using human THP-1 cells as described with modifications (34). THP-1 cells were stained with Hoechst 33342 live cell nuclear dye (catalog no.: C1025; Beyotime) at 37 °C for 15 min. Then the excess dyes were washed away by PBS, and cells were resuspended with serum-free culture medium. Subsequently, THP-1 cells at a concentration of 5 × 10^5^ cells/ml were added into ox-LDL-treated HUVECs. Afterward, Hoechst 33342-stained THP-1 cells and ox-LDL-treated HUVECs were cocultured for 6 h. Unadhered THP-1 cells were washed with PBS, followed by photographing with a DeltaVision microscopy system (100×, General Electric Company). The number of adhering THP-1 cells was calculated using Image J/Fiji.

### ELISA

Post treatment, the cultural supernatants of HUVECs in each group were collected and centrifuged at 1000 rpm for 2 min to remove cell debris. Supernatants were harvested for cytokine detection of MCP-1, sICAM-1, and sVCAM-1. The operation steps were strictly carried out according to the instructions of the ELISA kit (human MCP-1 ELISA kit: ab179886, Abcam; human sICAM-1 ELISA kit: 70-EK189-96, MultiSciences; human sVCAM-1 ELISA kit: DVC00, R&D Systems). In addition, ELISA was applied to detect M2 macrophage marker molecules (IL-10) and M1 macrophage marker molecules (IL-6 and TNF-α) in their corresponding supernatants, respectively, as per the instructions of the ELISA kit (human IL-10 ELISA kit: SEKH-0018; Solarbio; human IL-6 ELISA kit: SEKH-001; Solarbio; human TNF-α ELISA kit: SEKH-0047; Solarbio).

### qRT–PCR

Total RNA and miRNA were extracted from THP-1 cells and aortic arch of mice with the miRNeasy mini kit (catalog no.: 217004; Qiagen). TaqMan MicroRNA assay (catalog no.: 4427975; Applied Biosystems) with stem-loop RT primers was adopted for determining miR-147a expression. U6 snRNA was taken as an endogenous control.

For mRNA, RNA was reversely transcribed into complementary DNA using PrimeScript RT reagent Kit (catalog no.: RR037A; Takara) with random primers and oligo (dT). The expression of mRNA was measured with TB Green Premix Ex Taq II (catalog no.: RR820A; Takara). GAPDH was used as an endogenous control. The sequences of primer pairs are listed in Table 1. The relative expressions of genes were calculated by 2^−ΔΔCt^ method (35). The reactions in qRT–PCR were carried out in 7500 Fast Real-Time PCR System (Applied Biosystems).

**Table 1:** Primer sequences used in qRT–PCR

Gene	RT (5′→3′)	Forward (5′→3′)	Reverse (5′→3′)
miR-147a	GTCGTATCCAGTGCGTGTCGTGGAGTCGGCAATTGCACTGGATACGACGCAGAAGC	GTGGAAATGCTTCTGCGTCG	GTGTCGTGGAGTCGGCAATT
U6	CGCTTCACGAATTTGCGTGTCAT	CGCTTCACGATTTGCGTGTCAT	GCTTCGGCAGCACATATACTAAAAT
hsa-VLA4	Random hexamer	TATGCGGAAAGATGTGCG	GGCTGAAGTGGTGGGAAT
hsa-Mac-1	Random hexamer	TCCAGTGTGACATCCCGTT	GGTGGTTATGCGAGGTCTTG
hsa-CCR2	Random hexamer	ATGGTCATCTGCTACTCGGG	TGCCTCTTCTTCTCGTTTCG
hsa-ZEB2	Random hexamer	CAAGAGGCGCAAACAAGCC	GGTTGGCAATACCGTCATCC
hsa-GAPDH	Random hexamer	CACCACACTGAATCTCCCCT	TGGTTGAGCACAGGGTACTT
mmu-ZEB2	Random hexamer	AAACGTGGTGAACTATGACAACG	CTTGCAGAATCTCGCCACTG
mmu-CCR2	Random hexamer	ATCCACGGCATACTATCAACATC	TCGTAGTCATACGGTGTGGTG
mmu-Mac-1	Random hexamer	CCATGACCTTCCAAGAGAATGC	ACCGGCTTGTGCTGTAGTC
mmu-VLA4	Random hexamer	AACCGGGCACTCCTACAAC	CACCACCGAGTAGCCAAACAG
mmu-GAPDH	Random hexamer	AGGTCGGTGTGAACGGATTTG	GGGGTCGTTGATGGCAACA

### Western blot

The protein was isolated from cells and aortic arch of mice using radioimmunoprecipitation assay buffer (catalog no.: P0013B; Beyotime) and quantified with BCA assay kit (catalog no.: P0012S; Beyotime). Approximately 20 μg of proteins were isolated by electrophoresis on 5 to 12% SDS-PAGE gel using an SDS-PAGE Gel Preparation kit (catalog no.: P1200; Solarbio), with ColorMixed Protein Marker (catalog no: PR1910; 11–180 kDa) as a protein size marker. Then the proteins were transferred to Immobilon-P polyvinylidene fluoride membranes (catalog no.: YA1701; Solarbio). The membranes were subsequently exposed to primary antibodies (Table 2) and horseradish peroxidase–conjugated secondary antibodies (Table 2). Signals were detected with ECL Western Blotting Substrate (catalog no.: PE0010; Solarbio). Protein bands were quantified by ImageJ software with GAPDH as a loading control.

**Table 2:** List of primary antibodies used for Western blots

Protein	Host species	Catalog number	Company	Antibody dilution
CCR2 (human)	Rabbit	12199	CST	1:1000
CCR2 (mice)	Rabbit	ab203128	Abcam	1:1000
Mac-1	Rabbit	ab185723	Abcam	1:1000
VLA4	Rabbit	ab81280	Abcam	1:1000
ZEB2	Rabbit	ab191364	Abcam	1:1000
MHC-II (human)	Mouse	ab55152	Abcam	1:1000
MHC-II (mice)	Rabbit	ab180779	Abcam	1:1000
IL-1β	Mouse	12242	CST	1:1000
CD206	Rabbit	ab64693	Abcam	1:1000
Bcl-2	Rabbit	ab59348	Abcam	1:1000
Bax	Rabbit	ab32503	Abcam	1:1000
C caspase3	Rabbit	ab2302	Abcam	1:500
C caspase9	Rabbit	ab2324	Abcam	1:200
Arg-1	Rabbit	93668	CST	1:1000
GAPDH	Mouse	ab8245	Abcam	1:500
Secondary antibody	Goat Anti-Rabbit IgG H&L (HRP)	ab205718	Abcam	1:2000
Secondary antibody	Goat Antimouse IgG H&L (HRP)	ab205719	Abcam	1:2000

### Flow cytometry assay

Following treatment with M1 + MC, M2 + IC, M1 + M, or M2 + I conditioned medium, HUVECs were harvested for apoptosis (early and late) detection with Annexin V–FITC Apoptosis Detection Kit (catalog no.: C1062M; Beyotime). Briefly, HUVECs were collected through centrifugation (1000*g*, 5 min) and subsequently resuspended in Hank’s balanced salt solution. Next, 1 × 10^5^ resuspended cells were centrifuged again as aforementioned, and the cells were exposed to 195 μl Annexin V–FITC conjugation liquid, 5 μl Annexin V–FITC, and 10 μl propidium iodide solution for 10 min at 25 °C. The apoptotic rate was analyzed by the flow cytometer Accuri C6 with Cell Quest software (version no. 3.3; both BD Biosciences).

### ISH and immunofluorescence for cellular localization of miR-147a and ZEB2

ISH for miR-147a was performed conforming to the protocol of the miRCURY LNA miRNA ISH kit (catalog no.: 339450; hybridization buffer, Exiqon, Inc). In short, 7-μm-thick formalin-fixed and paraffin-embedded tissue sections were deparaffinized, rehydrated in a descending series of ethanol, and pretreated in a microwave with citrate buffer (catalog no.: ab64214; Abcam) for 20 min at high power. Subsequently, sections were thoroughly washed, placed in a humidified chamber, and prehybridized at 45 °C with 1× miRCURY LNA miRNA ISH (catalog no.: 339450; hybridization buffer, Exiqon Inc). A digoxigenin-labeled LNA-modified miR-147a DNA probe (catalog no.: 339112; Exiqon, Inc) was hybridized at 40 nM hybridization buffer at 50 °C overnight. Digoxigenin-labeled probes (*red*) were detected by Antidigoxigenin Fab antibody fragments conjugated with alkaline phosphatase (Roche Diagnostics Corporation). Immunofluorescence was used for detecting Mac-2 (rabbit anti-Galectin-3 antibody, 1 in 1000 dilution, catalog no.: YT0695, immunoway; Plano) and ZEB2 (catalog no.: YT4300, rabbit anti-ZEB2; 1 in 200 dilution; immunoway). Results were visualized under a fluorescent microscope after staining (Olympus BX 60 fluorescence microscope) at 100× and 400× magnifications. The positive cells were quantified in using ImageJ.

### Histology

Oil Red O staining was conducted for morphological examination of aortic arch. The aortic arch was cut into 10 serial 2.5 mm sections and snap-frozen in liquid nitrogen. The tissues were subsequently placed on a cryotome, and 10 μm serial sections of the aortic arch were harvested on coated glass slides. Oil Red O stains (catalog no.: O9755; Sigma–Aldrich) and hematoxylin–eosin (catalog no.: G1120; Solarbio) were used to dye every fifth section of the aortic arch, which were observed by a microscope and photographed under 40× or 100× magnification. The area of atherosclerotic plaque was measured in pixels using ImageJ. Masson staining was performed using Masson's Trichrome Stain Kit (catalog no.: G1340; Solarbio) to detect collagen content in the atherosclerotic lesions, and sections were photographed under 100× magnification.

### Data analysis

Statistical analyses were conducted with GraphPad Prism 8.0 (GraphPad Software, Inc). All assays in every group were performed in triplicate, and measurement data were expressed as mean ± SD. Two groups of data were compared using an independent samples *t* test. The one-way ANOVA was used to compare multiple groups, followed by post hoc Dunnett’s or Tukey’s test. Pearson’s correlation was performed among groups.

## Data availability

The analyzed datasets generated during the study are available from the corresponding author on reasonable request.

## Supporting information

This article contains supporting information.

## Conflict of interest

The authors declare that they have no conflicts of interest with the contents of this article.
