# Supplementary material for: miR-147a targets ZEB2 to regulate ox-LDL-induced monocyte adherence to HUVECs, atherosclerotic plaque formation, and stability in atherosclerosis
Source: J Biol Chem. 2023 Mar 29;299(6):104657. doi: 10.1016/j.jbc.2023.104657 (PMC10209027; doi:10.1016/j.jbc.2023.104657)

Supplementary figure 1. A flowchart of the treatments of HUVECs and THP-1 cells.

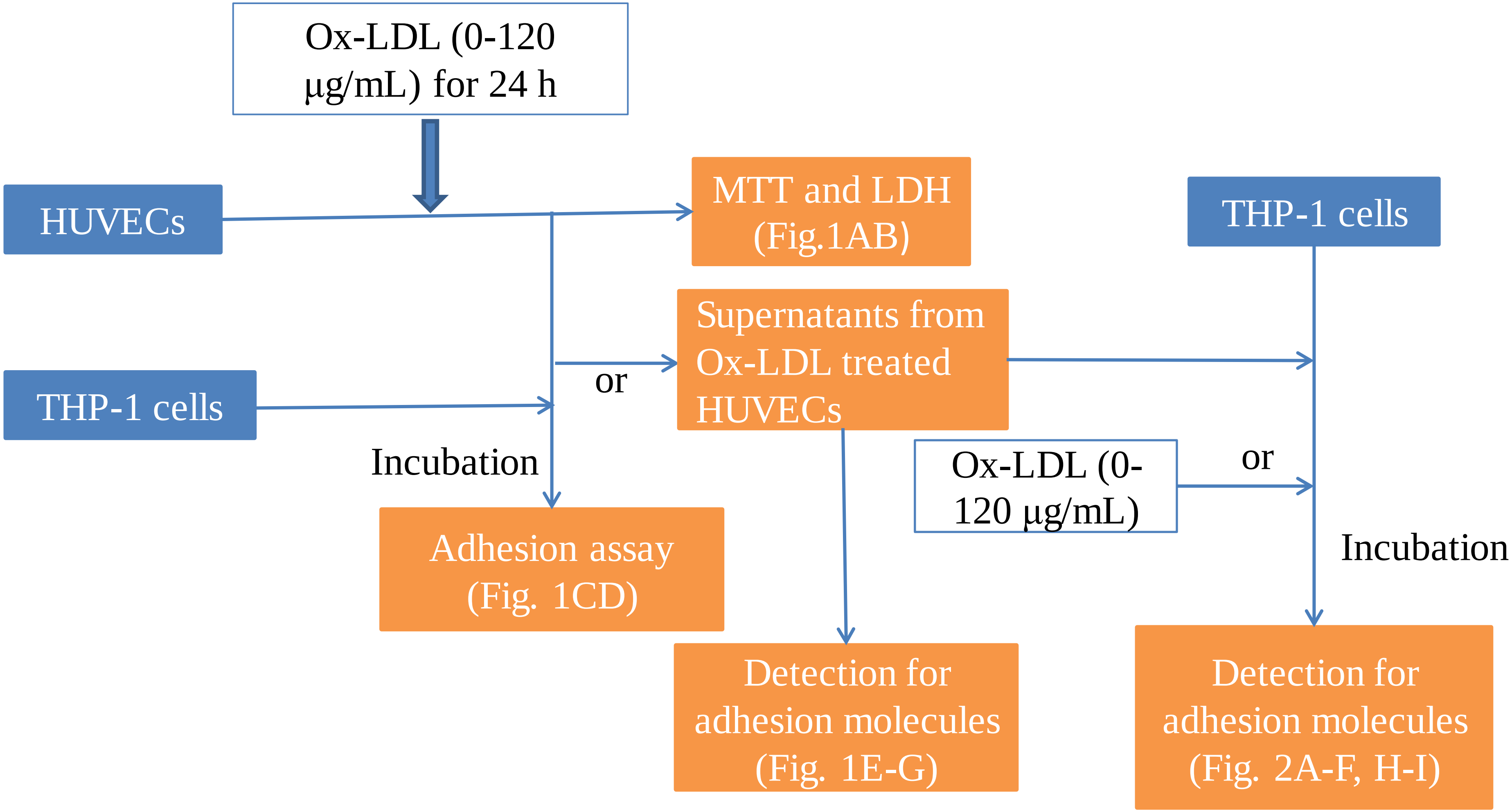

Supplementary figure 2. Ttransfection treatments of THP-1.

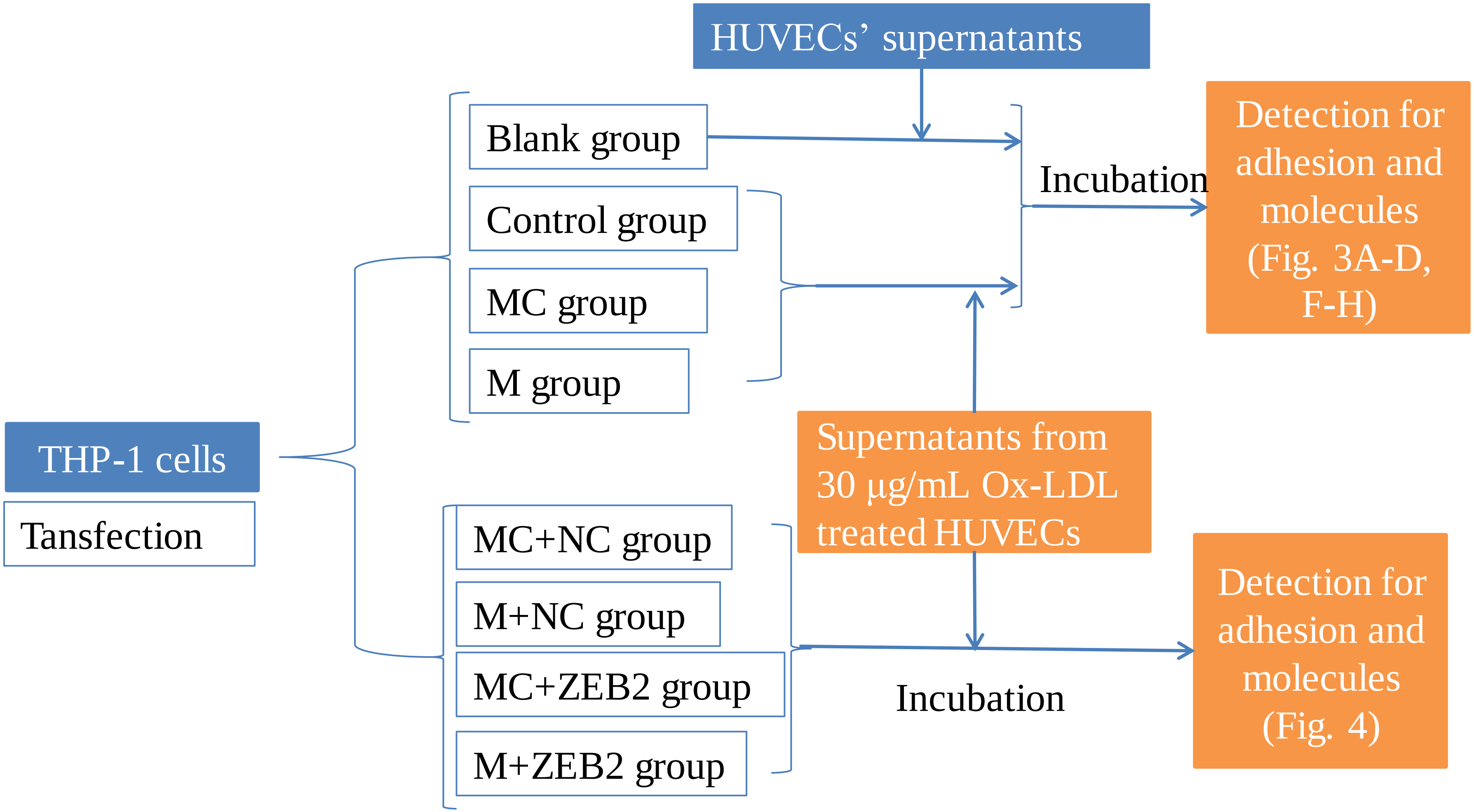

Supplementary figure 3. M1/M2 macrophages polarization of THP-1.

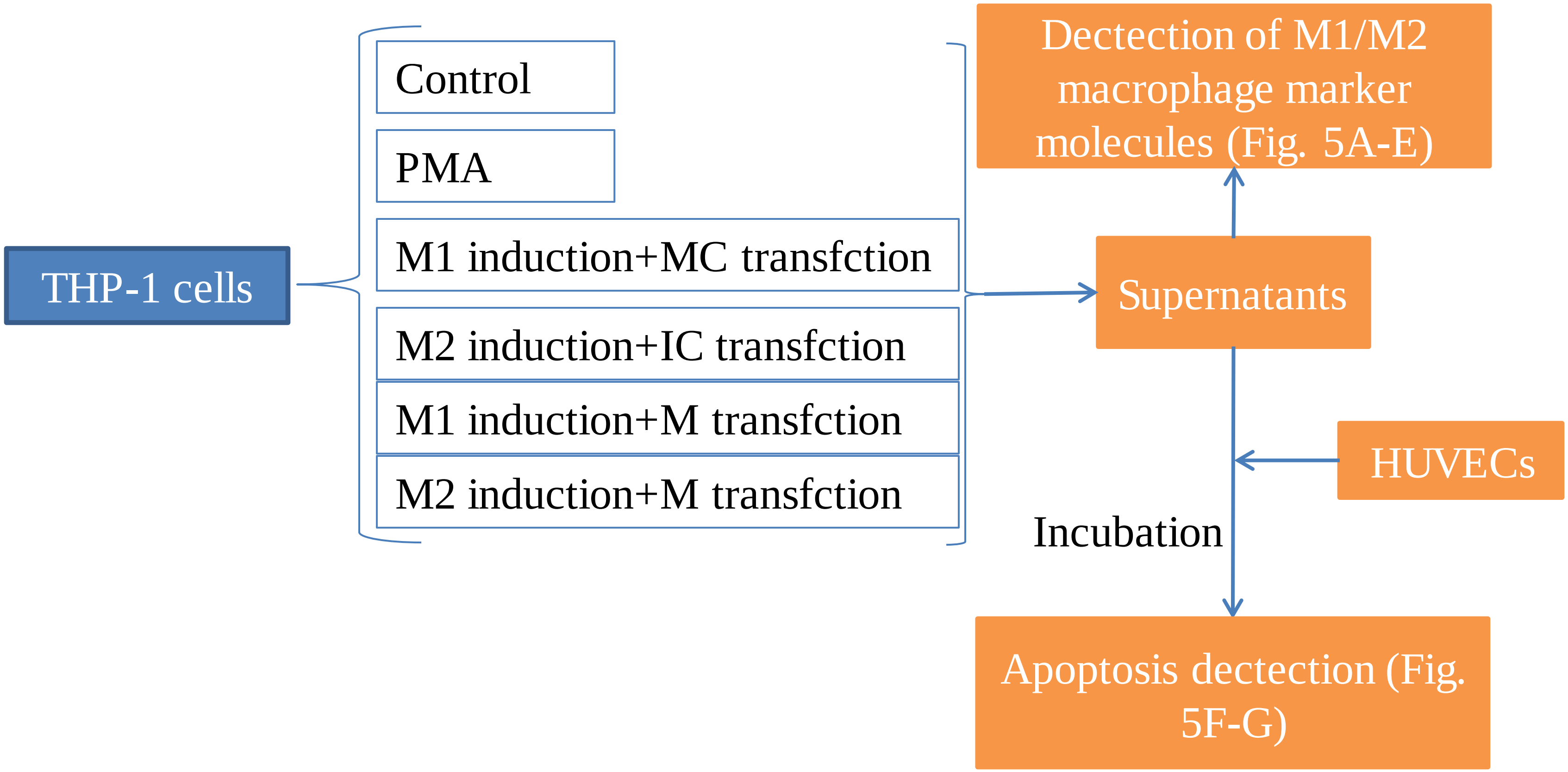

Supplement: Supporting Figures S1–S3 [file mmc1.pdf]
